# Differential uptake, kinetics and mechanisms of intracellular trafficking of next-generation antisense oligonucleotides across human cancer cell lines

**DOI:** 10.1093/nar/gkz214

**Published:** 2019-03-30

**Authors:** Emily Linnane, Paul Davey, Pei Zhang, Sanyogitta Puri, Mark Edbrooke, Elisabetta Chiarparin, Alexey S Revenko, A Robert Macleod, Jim C Norman, Sarah J Ross

**Affiliations:** 1 Bioscience, Oncology, IMED Biotech Unit, AstraZeneca, Cambridge, CB10 1XL, UK; 2 Chemistry, Oncology, IMED Biotech Unit, AstraZeneca, Cambridge, CB4 0WG, UK; 3 Advanced Drug Delivery, Pharmaceutical Sciences, IMED Biotech Unit, AstraZeneca, Cambridge, CB21 6GH, UK; 4 Ionis Pharmaceuticals, Carlsbad, CA 92010, USA; 5 Cancer Research UK Beatson Institute, Glasgow, G61 1BD, UK; 6 Institute of Cancer Sciences, University of Glasgow, Glasgow G61 1QH, UK

## Abstract

Antisense oligonucleotides (ASOs) modulate cellular target gene expression through direct binding to complementary RNA. Advances in ASO chemistry have led to the development of phosphorothioate (PS) ASOs with constrained-ethyl modifications (cEt). These next-generation cEt-ASOs can enter cells without transfection reagents. Factors involved in intracellular uptake and trafficking of cEt-ASOs leading to successful target knockdown are highly complex and not yet fully understood. AZD4785 is a potent and selective therapeutic *KRAS* cEt-ASO currently under clinical development for the treatment of cancer. Therefore, we used this to investigate mechanisms of cEt-ASO trafficking across a panel of cancer cells. We found that the extent of ASO-mediated *KRAS* mRNA knockdown varied significantly between cells and that this did not correlate with bulk levels of intracellular accumulation. We showed that in cells with good productive uptake, distribution of ASO was perinuclear and in those with poor productive uptake distribution was peripheral. Furthermore, ASO rapidly trafficked to the late endosome/lysosome in poor productive uptake cells compared to those with more robust knockdown. An siRNA screen identified several factors mechanistically involved in productive ASO uptake, including the endosomal GTPase Rab5C. This work provides novel insights into the trafficking of cEt-ASOs and mechanisms that may determine their cellular fate.

## INTRODUCTION

Antisense oligonucleotides (ASOs) are single-stranded DNA/RNA-like molecules that can be used as biological tools to modulate the expression of specific cellular target RNA. ASOs function through Watson–Crick hybridization, directly binding to complementary RNA sequences and modulating function. This is done through a number of different mechanisms, including recruitment of the enzyme Ribonuclease H (RNase H) that cleaves the RNA/ASO duplex leading to the down-regulation of target mRNA and protein (1,2). As ASOs are designed solely based on gene sequences they can be utilized to develop a wide range of inhibitors including those against previously ‘undruggable proteins’ that are difficult to target by classical therapeutic approaches. Recent progress in ASO chemistry has enabled the advancement of therapeutic ASOs with drug-like properties (3,4). These next-generation ASOs have chemical modifications including a phosporothioate (PS) backbone and 2′-4′constrained ethyl chemistry (cEt) at either end of the molecule. These modifications improve the potency of cEt-ASOs compared to the 2′-O-methoxyethyl (2′-MOE) oligonucleotides, and a number of cEt-ASOs are currently being developed in several disease areas including cancer (5–8).

cEt-ASOs are able to enter cells without the need of a delivery reagent in a process termed ‘free uptake’ that is mediated through context-dependent endocytic mechanisms (9–11). Pathways of ASO uptake resulting in target engagement are considered ‘productive’; however to date mechanisms of productive trafficking of ASOs have not been fully elucidated (12,13). ASO cellular internalization is known to be dependent on ASO binding with membrane-associated or extracellular proteins. Recent studies using 2′MOE-ASOs have begun to characterize cellular internalization mechanisms that may be important for productive uptake. For example, in mouse hepatic cells, uptake appears to be through a clathrin-independent but Adaptor-Related Protein Complex 2 Mu 1 Subunit (AP2M1)-dependent mechanism (14). Others have shown that Stabilin receptors bind ASOs with high affinity, and are responsible for bulk, clathrin-mediated endocytosis in mouse and rat liver cells (15). Another recent study in A431 cells showed that epidermal growth factor receptor (EGFR) binds ASOs at the cell surface and is important for productive ASO uptake through trafficking from early to late endosomes and may possibly contribute to the release of PS-ASOs from late endosomes (16). Once internalized, the ASO enters the endocytic network and is reported to distribute to early and late endosomes and also lysosomes (17). Escape from membrane-bound organelles is also considered to be important for the ASOs to engage with target mRNA and mediate a functional effect. Wang *et al.* characterized the importance of Annexin A2 (ANAX2) in facilitating endocytic trafficking of PS-ASOs modified with 2′-MOE chemistry, leading to target engagement and knockdown in HeLa and A431 human cell lines as well as mouse MHT cells via the release of ASOs from late endosomal compartments (18). Other data have shown that lysobisphosphatidic acid (LBPA) is required for the release of 2′MOE ASOs from the late endosome and subsequent activity on its mRNA target in the cell (19). Interestingly, across a panel of cell lines, the IC_50_ of target knockdown with an ASO applied without a delivery reagent can differ dramatically, which is likely due to differences in cellular uptake and trafficking of the ASO molecules. The mechanisms that mediate intracellular uptake that leads to productive target engagement by ASOs are clearly complex and, despite recent advances in the field, are not yet fully understood.

To date there have been no studies to characterize trafficking of cEt-ASOs within the cell and limited *in vitro* studies across ASO molecules in clinically relevant models. AZD4785, a potent and selective *KRAS* therapeutic cEt-ASO, is currently being developed to treat KRAS*-*dependent tumours. It is a 16-nt chimeric gapmer ASO that has PS linkages throughout the molecule and at either end has three nucleotides containing cEt modifications. AZD4785 binds to the 3′UTR of *KRAS* mRNA leading to the down-regulation of mRNA and protein and subsequent inhibition of downstream Ras effector pathways and proliferation in *KRAS-*mutant cells (20). AZD4785 was identified as a lead compound out of 2160 ASOs and was selected based on its activity in cells and xenograft models; systemic delivery of AZD4785 to mice bearing *KRAS-*mutant NSCLC xenografts or patient-derived xenografts resulted in inhibition of *KRAS* tumour expression and subsequent anti-tumour activity (20).

In this work, we sought to characterize pathways of cEt-ASO trafficking using AZD4785 across a panel of tumour cell lines with different levels of productive ASO uptake to provide a comprehensive overview of uptake and intracellular trafficking across clinically relevant cancer models. Understanding these mechanisms in relevant models could enable patient selection strategies and identify those most likely to benefit from cEt-ASO therapies. Furthermore, establishing relevant mechanistic pathways will enable identification of novel chemical, delivery and combination strategies to improve ASO productive uptake, reduce doses and cost of goods and increase translation to the clinic. Findings from our work highlight key cEt-ASO trafficking mechanisms, such as non-productive delivery to the late endosome, and influence of proteins including the endosomal Rab GTPase Rab5C that plays a crucial role in productive ASO trafficking within the cell.

## MATERIALS AND METHODS

### Cell lines and ASOs

MiaPaca-2, BxPC3, Panc 10.05, Colo-205, SW837, SW620, HT-29, NCI-H460, NCI-H1437, NCI-H358, A549, NCI-H2030, Calu-6, A427 and LK2 cell lines were from ATCC, PC9 were obtained from ECACC. All cell lines were authenticated by short tandem repeat (STR) analysis. Cells were cultured in RPMI media (Sigma) plus 10% foetal bovine serum (FBS). The antisense oligonucleotides used in this study were synthesized as described previously (4). The sequences of the ASOs used are AZD4785/Ionis 651987 GCTATTAGGAGTCTTT, Ionis 746275 TCTTGATTTGTCA*G*C*A* and Ionis 549148 GGCTACTACGCCGTCA. The letters indicate DNA, underlined letters indicate cEt modified bases and the italicized letters indicate MOE bases.

### ASO treatment

Cells between 20% and 50% confluency (depending on the timeframe of the assay) were dosed with ASOs 24 h after seeding. For cell dosing, ASOs were diluted in phosphate-buffered saline (PBS) before being administered to cells. Cells were then incubated at 37°C, 5% CO_2_ for the duration of the assay.

### RNA preparation, quantitative real-time PCR (RT-qPCR) and analysis

Total RNA was prepared from cells grown in 384-well plates or 96-well plates with FastLane Cell Probe kit or FastLane Cell Probe Multiplex kit (Qiagen). RNA lysates were used in real-time one-step RT-qPCR reactions performed on a Lightcycler 480 instrument (Roche). Gene expression values were calculated using the comparative delta delta *C*_t_ (-ΔΔ*C*_t_) method, using housekeeper gene *C*_t_ values and control-treated *C*_t_ for normalization as described previously (20). FAM MGB Assay Probes were obtained from Thermo Fisher Applied Biosystems: *KRAS* (Hs00364284_g1), *FOSL1* (Hs00759776_S1), *18S rRNA* (4352655), *RAB5C* (Hs00904926_g1), *RNASEH1* (Hs 007702360_s1), *KIF1A* (Hs00987720_m1), *KIF5C* (Hs00189672_m1), *RAB5A* (Hs00702360_s1) and *RAB5B* (Hs00951468_M1). For dual-colour RT-qPCR reactions, *18S rRNA* VIC (4318839) was used. For polymerase chain reaction (PCR) analysis of ASO dose response treatment, curves were plotted and data were analysed using non-linear regression and curves were fitted using log (inhibitor) versus response, variable slope (four parameters) equation using GraphPad Prism, curves were constrained between 0 and 1.

### Protein isolation and western blotting

For western blot analysis, cells were washed three times with ice-cold PBS and lysed in RIPA buffer (137 mM NaCl, 20 mM Tris–HCl, pH 7.4 10% (v/v) glycerol, 1% (v/v) Triton X-100 0.5% (w/v) sodium deoxycholate, 0.1% (w/v) SDS) supplemented with PhosSTOP Phosphatase Inhibitor Cocktail tablets (Roche), cOmplete Protease Inhibitor Cocktail tablets (Roche) and Benzonase Endonuclease (Merck Millipore). Protein lysates were separated on 4–12% Bis-Tris PAA gels (Invitrogen, ThermoFisher Scientific) and transferred to nitrocellulose membranes. Membranes were blocked in 5% w/v non-fat milk in PBS plus 0.05% v/v Tween 20 (PBST) for an hour and then probed with primary antibodies for 1 h at room temperature or overnight at 4°C. After washing, membranes were incubated with secondary antibodies coupled to horseradish peroxidase (HRP) for an hour at room temperature, washed and proteins were detected using chemiluminescence with SuperSignal West Dura Extended Duration Substrate (Thermo Fisher). Primary antibodies used for western blots were: KRas clone 2C1 (cat. C175665; Lifespan Bioscience); vinculin clone hVIN-1 (cat. V9131; Sigma); alpha-tubulin (DM1A, Cell Signalling Technology); Lamin B1 (Cell signalling technologies), secondary antibodies were sourced from Abcam (donkey anti-mouse- HRP, donkey anti-rabbit-HRP).

### Immunofluorescence

Cells were seeded onto 96-well glass-bottomed plates (Cellvis) at 70% confluency. The following day cells were fixed with 4% paraformaldehyde (PFA) (Affymetrix), washed three times in PBS and then permeabilised with 0.2% Triton X-100 (Sigma). Cells were blocked in 2% w/v bovine serum albumin (BSA) in PBS for 30 min before incubation with primary antibodies for 1 h at room temperature. Plates were then washed in PBS before being incubated with secondary antibodies for 1 h at room temperature in the dark. After washing again in PBS, cells were imaged using the Cell Voyager 7000 spinning disk confocal microscope (Yokogawa) using the 60× water objective. Primary antibodies used for immunofluorescence were: rabbit anti-ASO (Ionis 13545) (14), mouse anti-LAMP-1 (BD Bioscience), mouse anti-EEA-1 (BD Bioscience), Cis-Golgi (Abcam), mouse anti-alpha tubulin (DM1A, Cell Signalling) and rat anti-tubulin (Alexa Fluor® 647, Abcam). Secondary antibodies used were donkey anti-rabbit (Alexa Fluor® 488) and goat anti-mouse (Alexa Fluor® 555) sourced from Abcam. Nuclei were stained with Hoechst (Thermo Fisher).

### Image processing

All images were imported into Columbus analysis software (Perkin Elmer) for quantifications of cell nuclei, cell area, relative fluorescence (RFU) and co-localization. For analyses, cells were identified using Hoechst, anti-ASO antibody and tubulin stain, all boarder objects were removed and only full cellular objects (filtered on size) were used for further analysis. Cell area and morphology calculations were calculated as mean per well across a minimum of 16 fields per well. For co-localization analyses, preliminary validation of co-localization was first carried out in a small sample using FIJI (ImageJ) software (data not shown). Images were imported into Columbus software from one z-plane and correlation was calculated between the red and green channels using Pearson’s Correlation Co-efficient analysis. For RFU analysis, the quantification of AZD4785 fluorescence was determined in each cell and then corrected for the background from untreated controls. Data were expressed as mean per object (cell) per well, RFU was then divided by average cell area for each cell line. Representative images were compiled using FIJI (ImageJ) software from the original TIFF images.

### Mass spectrometry

NCI-H460 and LK2 cells were treated with 10 μM AZD4785. Cells were trypsinized, washed three times with ice-cold PBS and counted, before lysing in RIPA buffer as described previously. For cell fractionation experiments, cells were processed using the ThermoFisher subcellular fractionation kit (78840). Protein was quantified using BCA assay (Pierce). Samples were analysed by UPLC-MS utilizing a Waters Xevo TQ-XS (WBA0259) and an Acquity UPLC system from Waters consisting of Sample Manager (M16UFL953M), Acquity PDA (F17UPD457A), Column Oven (E17CMP703G) and Binary Solvent Manager (E17BUR621G). Full mass spectrometry optimization and methods can be found in Supplementary Figure S1.

### siRNA screening assay

Custom-made siRNA library assay plates were reconstituted according to manufacturer’s instructions (Dharmacon). NCI-H2030 cells were reverse transfected onto 384-well tissue culture plates (Perkin Elmer) using RNAi max (Invitrogen Thermofisher Scientific) at a final siRNA concentration of 20 nM. Cells were washed after 6 h and fresh media added. After 72 h, cells were treated with varying concentrations of AZD4785, left for 48 h before being processed for RT-qPCR as described previously. *KRAS* gene expression was normalized to housekeeping gene *18S rRNA* and shown relative to PBS control. Data were plotted using GraphPad Prism Software; for determining siRNA hits based on IC_50_, dose–response curves were plotted, and data were analysed using non-linear regression and curves were fit using log (inhibitor) versus response, variable slope (four parameters) equation using GraphPad Prism, curves were constrained between 0 and 1. For determining siRNA hits based on maximum inhibition, dose–response curves were plotted, and data were analysed using non-linear regression. For siRNA validation experiments, siRNA On-Target SMARTpool and single oligonucleotides were obtained from Dharmacon and reconstituted according to manufacturer’s instructions.

### Bioinformatic analysis

Oncoland Array Viewer was used to access TCGA patient data and CCLE_37, CCLE_38 and Sanger_38 cell line data. RNA Seq data were extracted and plotted using Graph Pad Prism or R Studio.

## RESULTS

### Differential productive uptake of AZD4785, a cEt-ASO, across tumour models *in vitro*

To investigate trafficking of cEt-ASOs within tumour cells, we firstly determined the sensitivity of different cancer cell lines from pancreatic, colon and lung tissue to cEt-ASO treatment. To do this we used AZD4785, an example of a next-generation ASO molecule with a phosphorothioate backbone and constrained ethyl chemistry, which targets the 3′UTR of *KRAS* mRNA (20).

The oncogene *KRAS* is reported to be required for the initiation of cancer and is associated with poor prognosis and poor patient survival (21,22). It is mutated in many cancers, including colorectal, pancreatic and non-small cell lung cancer (NSCLC) (23). Therefore, we constructed a cell line panel from these three tumour types and then employed *KRAS* mRNA knockdown as a measure of productive ASO uptake capacity across these cancer cells. Cell lines were selected to include both *KRAS*-wild-type and *KRAS*-mutant genotypes to determine any differences oncogenic KRas activity may have in mediating cEt-ASO uptake.

**Figure 1.**
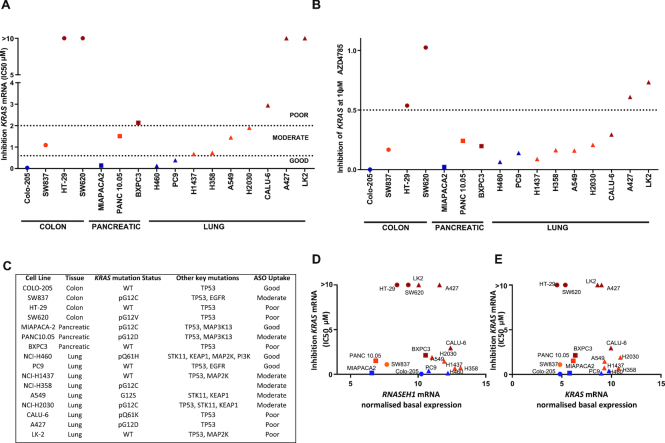
Differences in *KRAS* mRNA knockdown by cEt-ASO AZD4785 across a panel of cancer cell lines. (**A** and **B**) Colon, pancreatic and NSCLC cancer cells were treated with a dose range of AZD4785 for 72 h and RT-qPCR used to assess *KRAS* mRNA levels. Data were normalized to housekeeping gene *18S rRNA* and to PBS control. (A) IC_50_ and (B) maximum inhibition at 10 μM were calculated for each cell line from a minimum of two experiments. (**C**) Details of cell lines used in this study including key oncogenic mutations and productive uptake classification. (**D** and **E**) mRNA levels of (D) *RNASEH1* or (E) *KRAS* mRNA were determined by RT-qPCR on lysates from untreated cells. Data were normalized to the housekeeping gene *18S rRNA* and expressed as mean delta *C*_t_, then correlated with IC_50_ of inhibition of *KRAS* mRNA. Pearson’s correlation analysis was carried out on both *RNASEH1* and *KRAS* with IC_50_ of *KRAS* knockdown and was found to be non-significant (-0.1175 and -0.1904, respectively).

Cells were treated with ASO at doses ranging from 40 nM to 10 μM, *KRAS* mRNA levels were measured by RT-qPCR and IC_50_ and maximum inhibition at the highest ASO dose calculated (Figure 1A and B). We observed a range in response to ASO treatment across our cell panel that was not tissue dependent (Figure 1A and B; Supplementary Figure S2). Across the different cancer models, four out of sixteen cell lines: colorectal carcinoma cell line Colo-205, pancreatic cancer cell line MiaPaca-2 and lung cancer cell lines NCI-H460 and PC9 had robust knockdown of *KRAS* mRNA with a low IC_50_ (<600 nM) in response to AZD4785 treatment. Six out of sixteen cell lines: colorectal carcinoma cell line SW837, pancreatic cancer cell line Panc 10.05 and lung cancer cell lines NCI-H1437, NCI-H358, A549 and NCI-H2030 showed robust knockdown of *KRAS* mRNA but with moderate IC_50_ (>600 nM, <2 μM) and six out of sixteen cell lines: colorectal carcinoma cell lines SW620 and HT-29, pancreatic cancer cell line BxPC3 and lung cancer cell lines Calu-6, A427 and LK2 showed poor response to treatment with AZD4785, with little to no *KRAS* mRNA knockdown and high IC_50_ (>2 μM) (Figure 1A and Supplementary Figure S2). Although there was a continuum in free uptake capacity between cell lines, for the purposes of comparison, cell lines were then classified according to the degree of *KRAS* knockdown; good, moderate or poor with significant differences between the poor and other uptake groups (Supplementary Table S1). As mutant KRas has been reported to drive pathways of trafficking within the cell, including macropinocytosis and receptor-mediated endocytosis (24,25), we also assessed if there was any association between *KRAS* mutation status and cEt-ASO uptake. Surprisingly, there was no correlation between *KRAS* mutation status and AZD4785-mediated knockdown as both *KRAS-*mutant and wild-type genotypes span both good and poor productive uptake groups (Figure 1C). We next evaluated other key mutations associated with oncogenic and trafficking pathways; however, no correlation with the degree of ASO uptake was found (Figure 1C).

The mechanism of action by which ASO AZD4785 down-regulates *KRAS* mRNA is through RNase H1-mediated degradation. Thus, we assessed *RNASEH1* mRNA levels to determine whether differences in expression of *RNASEH1* could explain variation in productive ASO uptake. Relative basal levels of *RNASEH1* mRNA across the cell panel did not correlate with the extent of AZD4785-mediated knockdown observed (*r* = −0.1175), suggesting that this was unlikely to be the mechanism driving the differences in productive uptake (Figure 1D and Supplementary Figure S3). *KRAS* mRNA levels were also not correlated (*r* = −0.1904) with the degree of *KRAS* knockdown (Figure 1E and Supplementary Figure S4).

These data show that there are differences in productive ASO uptake leading to varying degrees of target knockdown across a diverse panel of tumour cell lines that are not dependant on total *RNASEH1* expression or basal levels of ASO target.

### cEt-ASOs enter cells in a dose-dependent manner with varied intracellular distribution in good and poor productive uptake models

**Figure 2.**
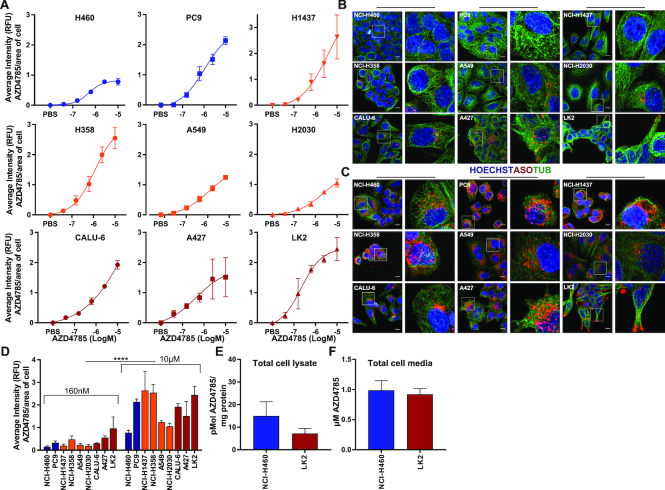
Quantification, intracellular distribution and accumulation of cEt-ASO across NSCLC cell lines. (**A**) Cell lines were seeded onto glass-bottomed plates and treated with a dose range of AZD4785 or PBS. Cells were fixed at 72 h and nuclei stained with Hoechst, cytoskeleton with alpha-tubulin and AZD4785 with an antibody that recognizes the phosphorothioate backbone of the antisense drug (anti-ASO antibody). Images were acquired and analysed using Columbus software. Hoechst was used to define the cell nucleus and alpha-tubulin and anti-ASO antibody to define the cell cytoplasm for Columbus analysis. Relative AZD4785 fluorescence (RFU) was quantified per object (cell) over 16 fields of view per well background corrected and normalized to the calculated average cell area of each cell line. Data were analysed from a minimum of three experiments, error bars represent standard error of the mean (SEM). (**B** and **C**) Representative images taken from NSCLC cells treated with (B) 160 nM or (C) 10 μM of AZD4785 for 72 h. Images were acquired and processed using FIJI (ImageJ) software; scale bar is 10 μm. (**D**) Relative ASO fluorescence was quantified over 16 fields of view per well and normalized to the average cell area of each cell line as previously described. Data were analysed from a minimum of three experiments, error bars represent SEM. Results showed significant increase in immunofluorescence between 160 nM and 10 μM dosing groups (*t*-test, *P*<0.001 (****)). (**E**) Mass spectrometry was carried out on lysates from LK2 and NCI-H460 cells dosed with 10 μM AZD4785 for 72 h to determine quantification of intact cellular ASO. Data were analysed from three independent experiments, error bars represent SEM. (**F**) Mass spectrometry was carried out on cell culture media from LK2 and NCI-H460 cell lines dosed with 10 μM AZD4785 for 72 h. Data were analysed from three independent experiments, error bars represent SEM.

As ASO-mediated *KRAS* mRNA knockdown varied across the cell line panel, we wanted to investigate if this was dependent on the amount of ASO internalized by the cell. To do this, we focused on NSCLC cells that are enriched for *KRAS* mutations occurring in ∼33% of lung adenocarcinoma (26). Cellular internalization of ASO was determined by immunofluorescence using an antibody that specifically detects the phosphorothioate backbone of the antisense drug (14,27). ASO accumulated within all the cells in a dose-dependent manner (Figure 2A and Supplementary Figure S5A), and even in cell lines with moderate or poor *KRAS* knockdown we observed ASO staining within the cell (Figure 2B and C). Kinetics of intracellular ASO accumulation was also evaluated and ASO-positive vesicles were detected within cells 30 min after treatment, indicating the AZD4785 ASO was internalized rapidly by endocytosis (Supplementary Figure S5B). ASO was detected within cells in vesicular structures at concentrations as low as 160 nM (Figure 2B and D), with ASO staining significantly increasing in the cell at 10 μM (Figure 2C and D). Interestingly, intracellular ASO staining showed distinct patterns of distribution across the different cell lines. LK2 and A437, poor productive uptake cells, had greater peripheral accumulation of ASO close to the cell membrane. In contrast, within good and moderate uptake cell lines there was greater ASO accumulation in the perinuclear region (Figure 2C). Mass spectrometry has been shown as an effective label-free method to measure intracellular exposure in cells of small molecule drugs (28). We extended the approach to measure cellular exposure of ASOs quantifying the amount of intact and labelled free intracellular AZD4785, focusing on NCI-H460 (IC_50_ < 150 nM) and LK2 (IC_50_ > 10 μM) as representative good and poor productive uptake cell lines. Mass spectrometry analysis showed there was no significant difference in total cellular intact AZD4785 in NCI-H460 cells and LK2 cells indicating similar levels accumulate in the cell (Figure 2E). Depletion of free ASO from the cell culture media was also quantified by mass spectrometry, with no significant difference detected between NCI-H460 and LK2 cells (Figure 2F). The amount of intact free ASO detected in cell media was low (0.98 and 0.92 μM, respectively) most likely due to ASO-protein binding in the media serum.

In summary, these data indicate that total levels of cEt-ASO accumulation within the cell do not correlate with target knockdown and that comparable levels of intact cEt-ASO are measured in total cell lysates from both good and poor uptake cell lines. However, there are differences in localization of cEt-ASO between these cell lines suggesting differences in intracellular pathways of trafficking.

### Productive uptake and distribution of additional ASOs

**Figure 3.**
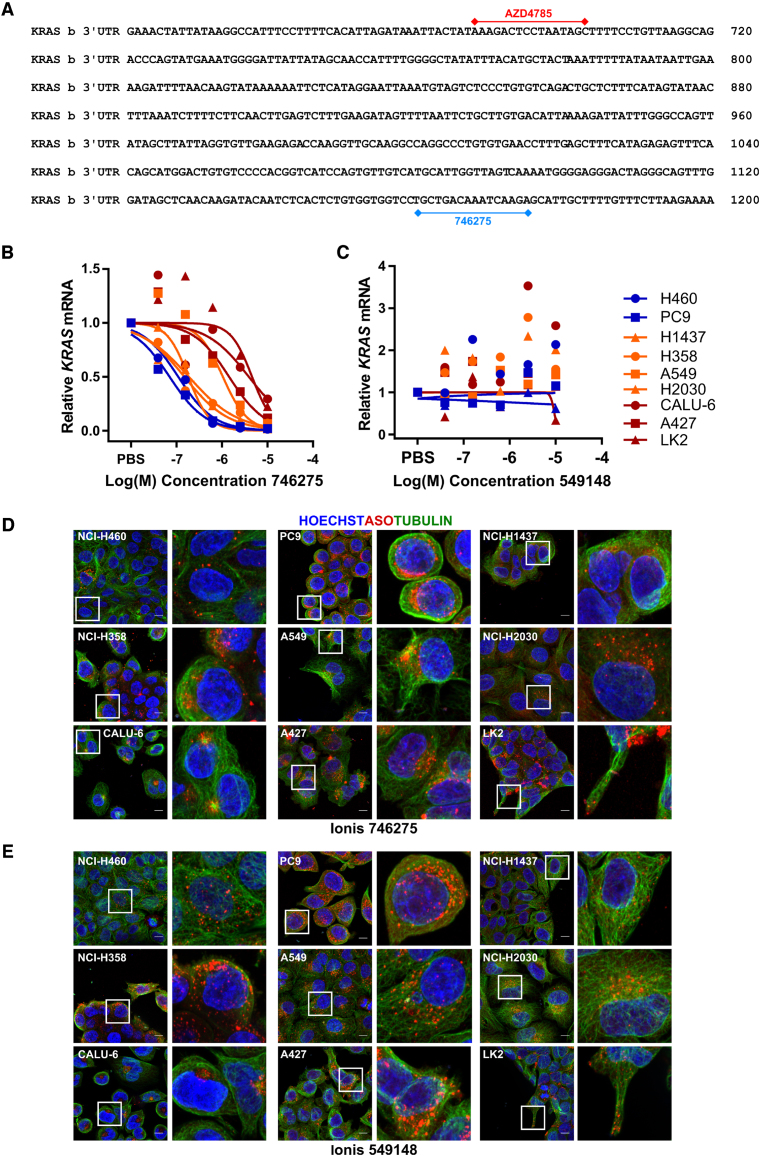
Uptake and intracellular distribution of additional ASO molecules across NSCLC cell lines (**A**) AZD4785 and Ionis 746275-binding sites on *KRAS* mRNA. (**B** and **C**) NSCLC cell lines were treated with a dose range of (B) Ionis 746275 or (C) Ionis 549148 for 72 h and RT-qPCR used to assess *KRAS* mRNA levels. Data were normalized to housekeeping gene *18S rRNA* and to PBS control. Data shown are representative of multiple experiments. (**D** and **E**) Cell lines were seeded onto glass-bottomed plates and treated with 10 μM of (D) Ionis 746275 or (E) Ionis 549148. Cells were fixed at 72 h and nuclei stained with Hoechst, cytoskeleton with alpha-tubulin and ASO with anti-ASO antibody, and images were processed using FIJI (ImageJ) software; scale bar is 10 μm.

To determine if the observation between ASO uptake and intracellular distribution is similar across other molecules, and not just specific to AZD4785, we expanded our study. We selected two additional ASOs; Ionis 746275, another *KRAS* targeting ASO that has both 2′MOE and cEt modifications (Figure 3A) and Ionis 549148, a non-targeting control ASO. We found that ASO-mediated *KRAS* knockdown by Ionis 746275 showed the same trends as AZD4785, with the panel of cell lines separating into similar groups of good (NCI-H460 and PC9), moderate (NCI-H1437, NCI-H358, A549 and NCI-H2030) and poor uptake cells (Calu-6, A427 and LK2) (Figure 3B). We then looked at the non-targeting ASO control, Ionis 549148 and RT-qPCR confirmed no target engagement with *KRAS* (Figure 3C).

We also compared intracellular distribution of these ASOs: Ionis 746275 showed similar cellular distribution to AZD4785, with perinuclear staining being observed in the cell lines with good ASO uptake, and more peripheral staining being observed in the poor uptake LK2 cell line (Figure 3D). The intracellular distribution patterns of the non-targeting control ASO, Ionis 549148, also showed the same trend as observed with the other ASOs (Figure 3E). These data show that the intracellular localization of ASOs is the same regardless of target or sequence suggesting that advanced chemistry ASOs all traffic in the cell in similar ways and probably through similar mechanisms.

### Kinetics of ASO-mediated *KRAS* knockdown varies between NSCLC cell lines

**Figure 4.**
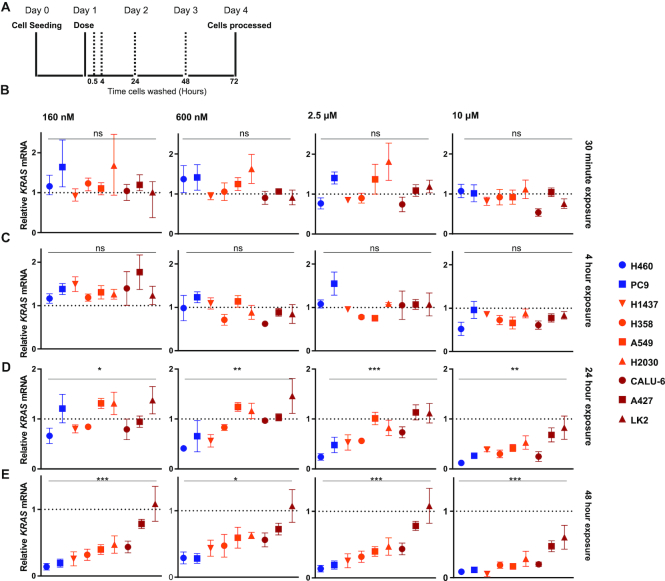
Kinetics of knockdown by cEt-ASO AZD4785. (**A**) Schematic showing the experimental design of this study. NSCLC cell lines were treated with a dose range of AZD4785 and washed after various time points before processing at 72 h. (**B**–**E**) Relative expression of *KRAS* mRNA by RT-qPCR after cells were exposed to a dose range of AZD4785 for (B) 30 min, (C) 4 h, (D) 24 h, (E) 48 h before samples were collected at 72 h after the start of initial dosing. Data were normalized to the housekeeping gene *18S rRNA* and shown relative to PBS control. Data represent the mean and range from a minimum of three independent experiments and error bars represent SEM. A one-way analysis of variance (ANOVA) was performed to assess significance, *P*<0.05 (*), *P*<0.01 (**) and *P*<0.001 (***). Tukey’s post-hoc test was carried out for multiple comparisons between cell lines (Supplementary Table S2).

Although internalization of ASO occurs rapidly, within 30 min of incubation (Supplementary Figure S5B), maximum target knockdown occurs later between 24 and 72 h post initial dosing depending upon the cell line. Therefore, to further explore the kinetics of ASO intracellular trafficking, we carried out pulse-chase experiments. Cells were treated with various doses of ASO for fixed exposure times, before washing and then incubating in fresh medium until processing at 72 h after the start of the treatment (Figure 4A).

A 30-min and 4-h pulse with ASO did not cause a significant reduction in *KRAS* mRNA expression in any of the cell lines at any concentration, indicating that longer exposure to the treatment is needed for target knockdown (Figure 4B and C). Following 24 and 48 h ASO exposure, levels of *KRAS* knockdown varied across cell lines. As a group the good productive uptake cell lines, NCI-H460 and PC9, showed *KRAS* knockdown with concentrations of AZD4785 as low as 600 and 160 nM; however, as expected, we do not observe this in poor cell lines A427 and LK2 (Figure 4D and E; Supplementary Table S2). At higher doses of AZD4785, we also see differences between the cell line groups; at 24-h exposure to 2.5 μM, there are significant differences between cell lines in the good uptake group compared to the moderate and poor uptake cell lines (Figure 4D and Supplementary Table S2). Together, these data highlight fundamental differences in the kinetics of productive uptake of ASOs across cell lines suggesting that the pathways of trafficking differ between these cell lines.

### Poor productive uptake cell lines show stronger cEt-ASOs co-localization with the late endosome/lysosome marker LAMP-1

**Figure 5.**
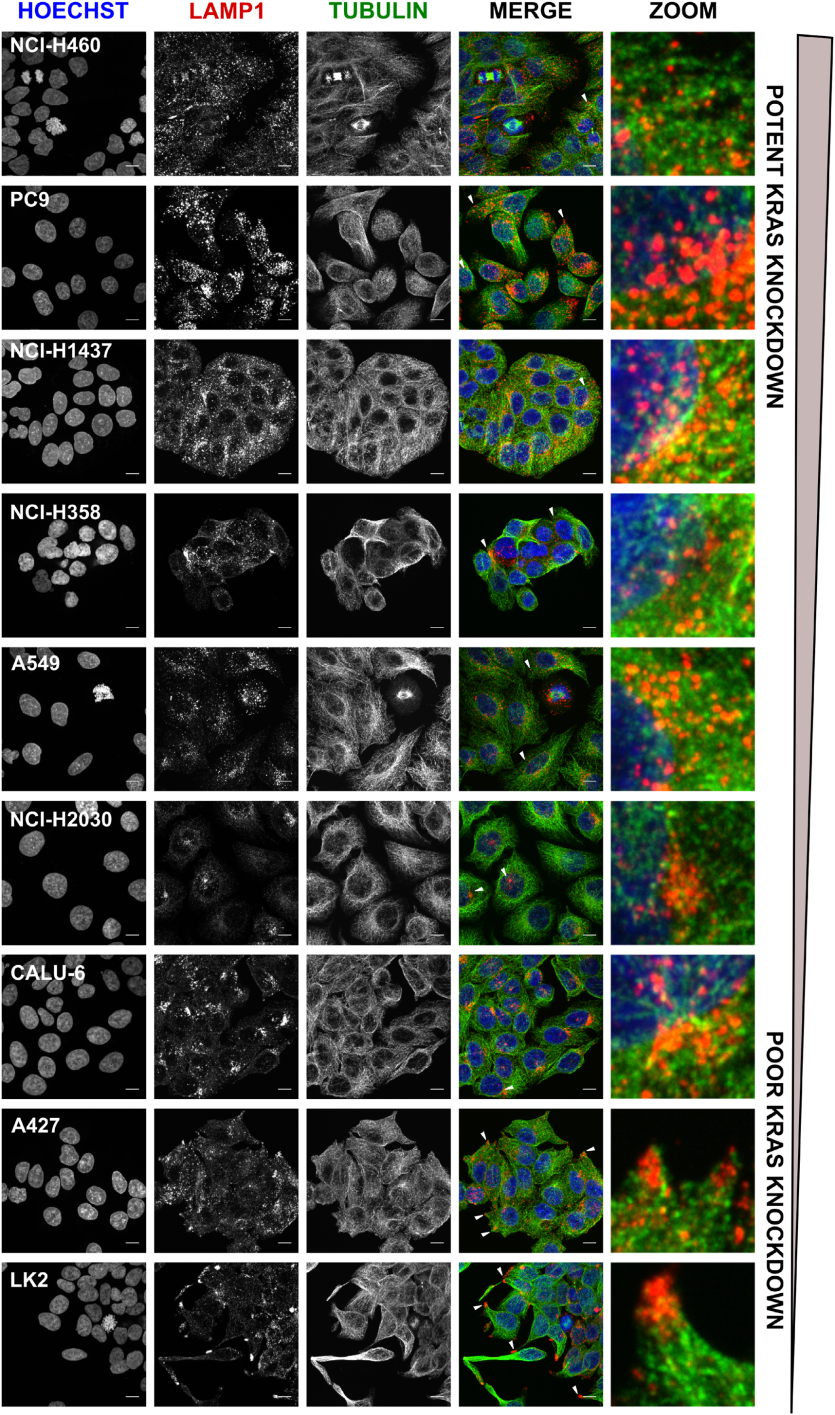
Lysosomal distribution is varied across the panel of cell lines. Confocal z-stack images of untreated NSCLC cells were acquired to visualize LAMP-1 across cell lines. Nuclei were stained with Hoechst, cytoskeleton with alpha-tubulin and late endosomes/lysosomes with LAMP-1. Images were processed using FIJI (ImageJ software) and shown as a maximum projection of the z-stack images. Lysosome distribution indicated on merged image by white arrows, zoom shows enlarged image section, scale bar 10 μm.

Previous work has established that ASOs enter cells through endocytic processes (13,14). Once internalized, they are trafficked to the early endosome and may have various fates including; being recycled back to the membrane through recycling endosomes, trafficking to the trans-Golgi network or transported to the late endosome to be delivered to the lysosome for degradation or into multivesicular bodies (MVBs) to be delivered out of the cell by exocytosis (29). Therefore, cell lines used in this panel were profiled to assess cell size and also the distribution of key cellular organelles involved in intracellular trafficking (Supplementary Figure S6). Early endosome antigen 1 (EEA1) was used to image early endosome localization, and EEA1 positive vesicles had distribution close to the perinuclear region as well as the cytoplasm across all cell lines (Supplementary Figure S7). Imaging of the late endosome and lysosome marker LAMP-1 showed distinct variations in cellular distribution across the cell lines (Figure 5). In the good productive uptake cell line, panel NCI-H460 and PC9 and also the moderate cell lines NCI-H1437, NCI-H358 and A549, LAMP-1 staining was scattered throughout the cytoplasm. However, in the NCI-H2030 and Calu-6 cell lines, LAMP-1 staining was shown to have a more endoplasmic reticulum-like distribution. Interestingly, in A427 and LK2 cell lines, which were shown to have the poorest productive uptake with no ASO-mediated *KRAS* knockdown, LAMP-1 staining localized to the cell protrusions and clustered close to the cell membrane (Figure 5).

**Figure 6.**
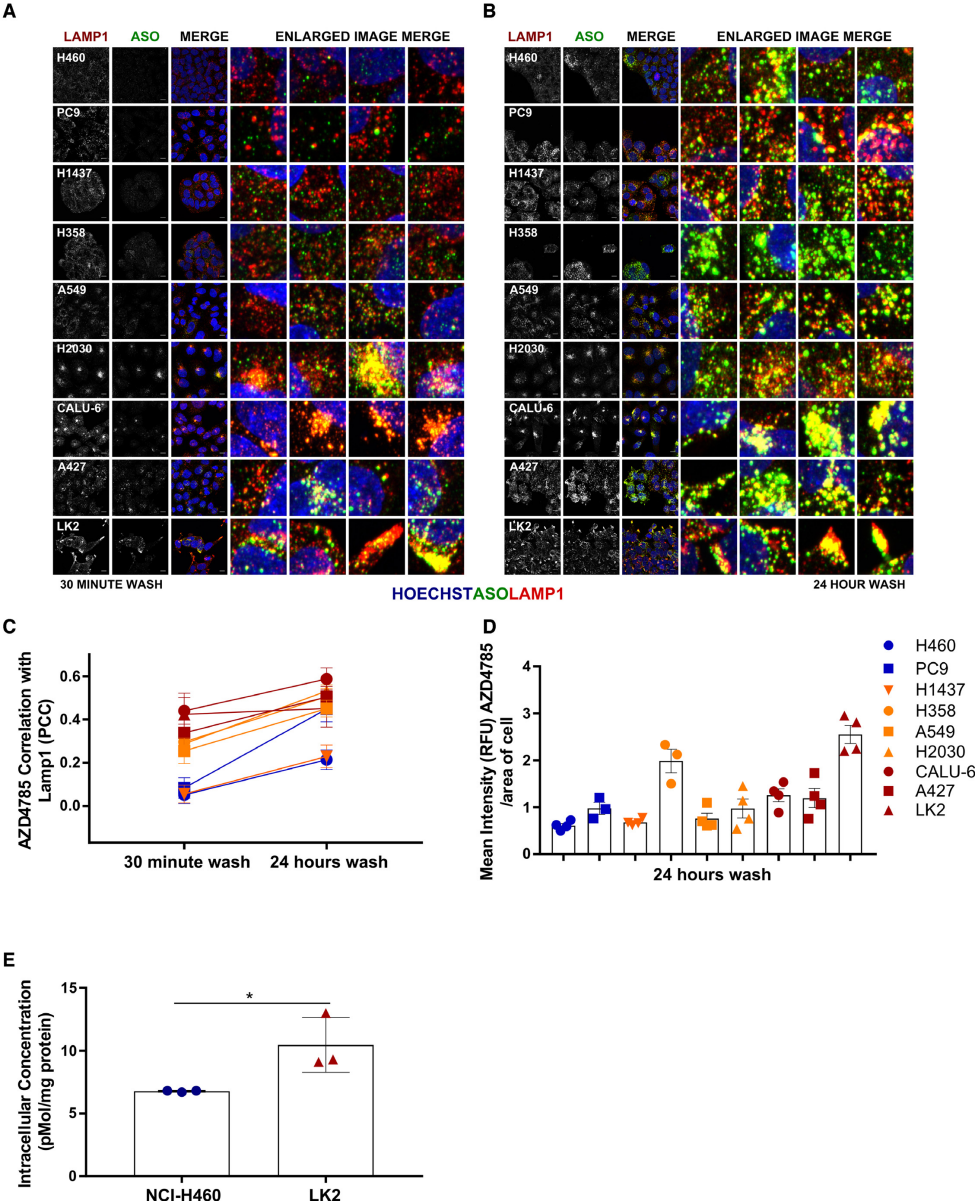
Differences in cEt-ASO trafficking to the lysosome across cell lines. Confocal z-stack images of NSCLC cells to visualize AZD4785 and LAMP-1 co-localization within the cell were taken 72 h after AZD4785 exposure (10 μM) for (**A**) 30 min and (**B**) 24 h, zoom shows 10× fold enlarged image section; scale bar 10 μm. (**C**) For co-localization quantification, confocal images were also taken on one z-plane 72 h after AZD4785 treatment. Co-localization between ASO and LAMP-1 in NSCLC cell lines was calculated by Pearson’s correlation coefficient (PCC) after 30 min or 24 h of AZD4785 exposure, on 16 fields of view per well, from a minimum of three independent experiments, error bars represent SEM. (**D**) Quantification of AZD4785 immunofluorescence in NSCLC cell lines after 24-h exposure to 10 μM of AZD4785, on 16 fields of view per well, from a minimum of three independent experiments, error bars represent SEM. (**E**) Mass spectrometry was carried out on lysates from NCI-H460 and LK2 cells to quantify intact AZD4785 oligonucleotides after 24-h exposure to 10 μM AZD4785, error bars represent standard deviation (SD). A one-way ANOVA was carried out to assess significance between groups, *P*<0.05 (*).

A series of co-localization experiments were set up to determine if there was any correlation of ASO staining with late endosome/lysosome markers. Again, we used a pulse-chase experiment design, where cell lines were treated with AZD4785 and washed at various time points before being fixed and stained for LAMP-1 and ASO at 72 h (Figure 6A and B). After 30-min exposure to 10 μM of AZD4785, co-localization between LAMP-1 and AZD4785 occurred in the poor uptake cell line cohort LK2, A427 and Calu-6 (Pearson’s correlation coefficient, PCC > 0.4). There was less correlation in the moderate cell lines NCI-H2030, NCI-H358 and A549 (PCC < 0.4, >0.2) and there was no correlation in co-localization in moderate cell line NCI-H1437 and good uptake cell lines NCI-H460 and PC9 (PCC < 0.09) (Figure 6C). After 24-h exposure to 10 μM of AZD4785, co-localization between LAMP-1 and AZD4785 increased across all cell lines, correlation was strong (PCC > 0.5) in the poor uptake cell line cohort (LK2, A427 and Calu-6). There was increased correlation in NCI-H2030, NCI-H358, A549 and PC9 cell lines (PCC < 0.5, >0.3); however, there was weaker correlation observed in the NCI-H460 and NCI-H1437 cell lines (PCC < 0.2) (Figure 6C).

We observed strong co-localization between ASO and LAMP-1 after cells were treated with 10 and 2.5 μM of ASO across all time points in poor uptake cell lines, suggesting rapid trafficking at higher doses of AZD4785 (Supplementary Figure S8). However, even treatment with lower concentrations of ASO lead to strong co-localization between LAMP-1 and ASO across the poor uptake cell lines at the later time points (24- and 48-h exposure) suggesting co-localization may also be time and dose dependent at lower concentrations in these cell lines (Supplementary Figure S8).

To determine how rapidly co-localization with LAMP-1 occurred, we also carried out a fixed time-course experiment in NCI-H460 and LK2 cells quantifying co-localization of ASO with LAMP-1 at 4 and 72 h following dosing (Supplementary Figure S9). Four hours following dosing with AZD4785, LK2 cells had stronger correlation of ASO and LAMP-1 co-localization at higher concentrations, compared to NCI-H460 (Supplementary Figure S9A and C). After 72 h treatment at concentrations from 40 nM AZD4785 and higher, we saw significantly higher co-localization of ASO with LAMP-1 in LK2 cells that we did not see in NCI-H460 cells (Supplementary Figure S9B and D). Thus, these data suggest that in NSCLC cell lines that have poor ASO-mediated *KRAS* knockdown, more ASO is being trafficked on the late endosome-lysosome pathway for degradation or exocytosis, which may be an important mechanism that prevents productive uptake of ASOs.

We also observed that there were some differences in general levels of AZD4785 detectable by immunofluorescence across cell lines following 24-h exposure, especially between NCI-H460, which has the best productive uptake and LK2, which has the worst (Figures 6D). To examine this more closely, mass spectrometry analyses were performed on total cell lysates from NCI-H460 and LK2 exposed to AZD4785 for 30 min or 24 h before being processed at 72 h (Figure 4A). A 30-min exposure to treatment resulted in ASO levels that were outside the low range in both cell lines (data not shown); however, after 24-h exposure, we detected higher levels of intact ASO within LK2 cells compared to NCI-H460 cells (Figure 6E). Robust *KRAS* knockdown occurs in NCI-H460 cells following 24 h exposure (Figure 4D); therefore the lower levels detected in these cells by mass spectrometry following the same exposure to AZD4785 could be due to degradation of cytoplasmic ASO or exocytosis from the cell following its activity.

### Kinesin-1 and Kinesin-3 Motor proteins are associated with ASO trafficking in poor productive uptake cells

**Figure 7.**
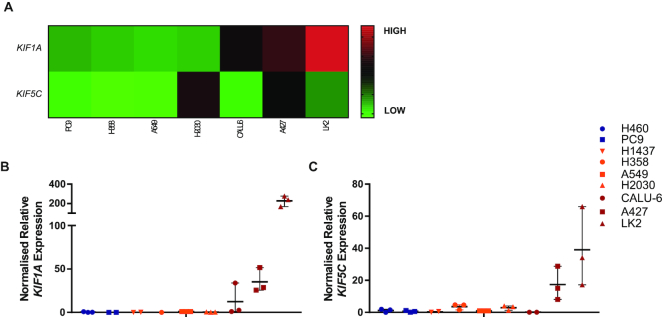
Expression of kinesin motor proteins across the cell panel. (**A**) Expression of *KIF1A* and *KIF5C* analysed using OmicSoft ArraySuite, OncoLand software, using RNAseq expression data for available cell lines exported from the Sanger_38 database. Log_2_ (FPKM+0.1) values were plotted as a heat map. (**B** and **C**) mRNA levels of *KIF1A* and *KIF5C* were determined by RT-qPCR on untreated cell lines, data were normalized to the housekeeping gene *18S rRNA* and shown relative to A549 cells, individual values from a minimum of three independent experiments are shown. Error bars represent the mean and the range.

To identify factors that may contribute to regulating differences observed in intracellular trafficking and lysosomal distribution of ASOs across models, a bioinformatics approach was utilized. Analysis of gene signatures associated with cellular trafficking was assessed for differential expression across the cell panel. Investigation of the kinesin and dynein gene families showed that genes (*KIF1A* and *KIF5C*) encoding components of the kinesin motor protein complexes, kinesin-1 and kinesin-3, were differentially regulated in poor productive uptake cell lines (Figure 7A and Supplementary Figure S10). RT-qPCR confirmed *KIF1A* mRNA levels were up-regulated in Calu-6, A427 and LK2 cells (Figure 7B), and *KIF5C* mRNA levels were up-regulated in A427 and LK2 cells (Figure 7C). These data show high levels of *KIF1A* and *KIF5C* expression correlate with poor productive ASO uptake in NSCLC cells, highlighting a potential novel pathway regulating non-productive ASO cellular trafficking.

### cEt-ASO accumulates in the nucleus of cells with productive uptake

**Figure 8.**
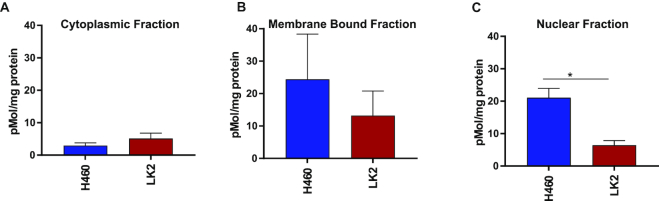
Detection of intact ASO from cellular fractions. Mass spectrometry was carried out on cellular fractions to quantify intact ASO after 72-h exposure to 10 μM AZD4785. (**A** and **B**) There were no significant differences between ASO in cytoplasmic and membrane fractions from three independent experiments (error represents SEM). (**C**) There was a significant difference between intact ASO in the nuclear fraction between the two cell lines from three independent experiments, (error bars represent SEM, a one-way ANOVA was carried out to assess significance between groups, *P*<0.05 (*)).

To further address where ASOs are trafficked within cells, additional mass spectrometry experiments were carried out using representative good (NCI-H460) and poor (LK2) cell lines dosed with 10 μM AZD4785 and fractionating into cytoplasmic, membrane bound and nuclear components. Mass spectrometry analysis showed that there were no significant differences between the cell lines for the cytoplasmic and membrane fractions (Figure 8A and B). However, analysis of the nuclear fraction showed that there were significantly higher levels of ASO in NCI-H460 cells, compared to LK2 cells (Figure 8C and Supplementary Figure S11) suggesting in good uptake cell lines ASO traffics to the nucleus.

### Productive ASO uptake is modulated by Rab5C

**Figure 9.**
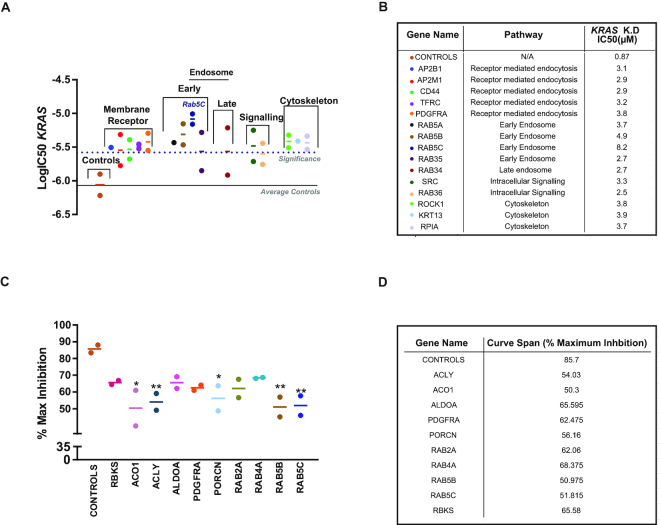
siRNA screen to identify factors that impact ASO productive trafficking. NCI-H2030 cell lines were transfected with siGenome pools and treated with a dose range of AZD4785, before RT-qPCR was carried out to determine *KRAS* mRNA levels. Data were normalized to housekeeping gene *18S rRNA* and PBS control. IC_50_ data were plotted and a 3-fold threshold cut off was applied to determine genes of interest compared to the non-targeting and RISC-free controls. (**A** and **B**) Genes that significantly change the IC_50_ of AZD4785 productive uptake. Data shown represent the mean IC_50_ and range of biological duplicates from two independent screening experiments. (**C** and **D**) Maximum % inhibition of KRAS mRNA following treatment with AZD4785. Data represent the mean and range of biological duplicates from two independent screening experiments, a one-way ANOVA was used to calculate significance compared to the controls, indicated by the asterisk (* *P*<0.05, ** *P*<0.01).

To elucidate additional pathways and functional factors that could be responsible for the variations observed in productive uptake and in cellular distribution and trafficking of ASOs, we carried out a gene silencing (siRNA) screen. As trafficking of ASOs had previously been described as an endocytic process (9–11), the screen was carried out using a library of 210 genes involved in membrane trafficking. Genes involved in metabolic processes and translation have also been linked to the productive uptake of ASOs; therefore, we included a selection of genes involved in regulating metabolic pathways (30–32). We used a siRNA screening approach, as siRNA act to silence mRNA via incorporation with RNA-induced silencing complex (RISC) and not an RNase H-dependent mechanism, so, therefore, would not interfere with AZD4785 antisense oligonucleotide activity. NCI-H2030 cells, a moderate uptake line, were selected to be used in this screen as we reasoned this would enable the detection of factors that either increase or decrease productive ASO uptake. Cells were transfected with siRNA and then treated with a dose response of AZD4785 to determine the effect on ASO-mediated *KRAS* down-regulation. Data were analysed by looking for changes in the IC_50_ (Figure 9A) and maximum inhibition of *KRAS* mRNA (Figure 9C) following ASO treatment. A number of genes had an impact on ASO-mediated *KRAS* knockdown when silenced. The screening hits which decreased ASO IC_50_ were ranked and the highest ranking genes were taken forward for further validations; the *RAB5* isoform, *RAB5C*, was the top-ranking hit from the screen (Figure 9B and D).

**Figure 10.**
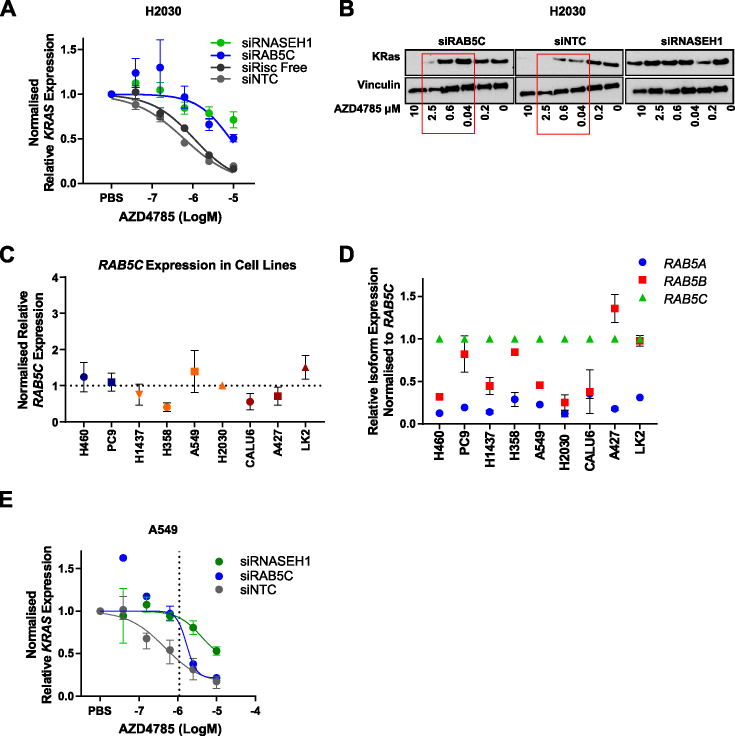
*RAB5C* regulates ASO productive uptake in NSCLC. (**A**) Data showing IC_50_ dose–response curves for *KRAS* mRNA knockdown in H2030 cells transfected with siRNA targeting *RNASEH1, RAB5C* or negative controls for 72 h and then treated with AZD4785 for 48 h. Data were normalized to housekeeping gene *18S rRNA* and PBS control. Results were expressed from the mean of biological duplicates from each experimental run and then averaged across two independent experiments. Error bars represent SEM. (**B**) Western blot analysis of KRas and vinculin levels in lysates from H2030 cells transfected for 72 h with siRNA On-Target SMARTpools for *RAB5C, RNASEH1* or non-targeting control (siNTC) before treating with AZD4785 for 48 h. Red box highlights differences in KRas proteins levels between control and siRAB5C transfected cells. (**C**) RT-qPCR was carried out on untreated cells to determine expression levels of *RAB5C* across the cell panel. Data were normalized to *18S rRNA* control and shown relative to H2030 cells. Data shown represent the mean and SEM from a minimum of three experiments. (**D**) RT-qPCR was carried out on untreated cells to determine expression levels of *RAB5* isoforms across the cell panel. Data were normalized to *18S rRNA* control and shown relative to *RAB5C*. Data shown represent the mean and SD from a minimum of two experiments. (**E**) Data showing IC_50_ dose–response curves for *KRAS* mRNA knockdown in A549 cells transfected with siRNA targeting *RNASEH1, RAB5C* or siNTC for 72 h and then treated with AZD4785 for 48 h. Data were normalized to housekeeping gene *18S rRNA* and PBS control. Results were expressed from the mean of biological duplicates from an experimental run, error bars represent the range.

Knockdown of Rab-GTPase *RAB5C* using a siRNA pool prior to dosing with AZD4785 caused a shift in the IC_50_ of *KRAS* mRNA knockdown from 870 nM to 8 μM, indicating this is required for the productive trafficking of the drug (Figure 10A). The importance of *RAB5C* in the productive uptake of ASOs was also confirmed using individual siRNA (Supplementary Figures S12 and S13). Western blotting also confirmed that siRAB5C impaired ASO-mediated knockdown of KRas protein compared to the control siRNA (Figure 10B). We assessed whether there was any association between degree of ASO uptake (as measured by *KRAS* knockdown) and *RAB5C* expression across our panel of NSCLC cell lines; however there was no correlation (Figure 10C). *RAB5* has three isoforms: A, B and C, and data from our siRNA screen also identified *RAB5A* and *RAB5B* as important in ASO productive uptake (Figure 9). However, *RAB5C* appears most important in NCI-H2030 cells. We examined expression of the*RAB5* isoforms across the panel of cell lines by RT-qPCR and found that in NCI-H2030 cells the *RAB5C* isoform was more highly expressed than *RAB5A* and *RAB5B* (Figure 10D). This trend was also observed in NCI-H460, NCI-H1437, A549 and Calu-6 cells (Figure 10D) and analysis of data from the Cancer Genome Atlas also confirmed this trend across patient samples (Supplementary Figure S14). However, PC9, NCI-H358, A427 and LK2 cells showed similar expression levels of *RAB5B* to *RAB5C* (Figure 10D). Next, we wanted to confirm if *RAB5C* depletion impacted ASO-mediated *KRAS* knockdown across other moderate uptake cell lines. We found that siRAB5C affected *KRAS* knockdown in A549 cells, another moderate uptake cell line (Figure 10E) although no impact was seen in NCI-H358 cells (data not shown), perhaps due to high relative co-expression of *RAB5B* (Figure 10D). Taken together these data suggest that Rab5 proteins, in particular Rab5C, are important in regulating productive ASO trafficking.

## DISCUSSION

Advances in ASO chemistry have enabled the generation of therapeutic ASOs with drug-like properties including AZD4785, a potent and selective *KRAS* ASO that is being developed to treat KRas*-*dependent tumours (20). Despite progress in the field of ASO trafficking and delivery, productive uptake of advanced chemistry ASOs is a complex process that is not yet fully understood (17,33). Understanding the mechanisms of ASO trafficking in relevant tumour models could enable insights into medicinal chemistry, delivery and combination approaches to improve ASO productive uptake, as well as identifying patient selection strategies to ascertain those most likely to benefit from antisense therapeutics such as AZD4785.

### ASO productive uptake varies within tumour models but does not correlate with quantity of internalized ASO

We examined the mechanisms of ASO trafficking across a tumour cell line panel to provide an overview of uptake and intracellular trafficking across clinically relevant models. Here, we report in colon, pancreatic and NSCLC cell lines, the IC_50_ of target knockdown with AZD4785 (with unformulated delivery) differed dramatically, ranging from cell lines that had a robust knockdown, to those that had minimal or no knockdown of *KRAS* mRNA. These differences were observed across cell lines and did not correlate with basal expression of *RNASEH1* or *KRAS* mRNA, indicating that pathways of ASO trafficking or mechanisms of endosomal release must be differentially regulated across cell lines. Immunofluorescence and mass spectrometry both confirmed that similar levels of ASO enter the cell, regardless of the degree of target knockdown, highlighting the importance of ASO intracellular trafficking.

### Rab5C plays a role in productive ASO trafficking

It is known that ASOs enter cells through endocytic events and are delivered to the early endosome (9). The early endosome (also referred to as the sorting endosome) plays a crucial role in endocytosis as it determines the route of cargo (34). Material can be transported from early to late endosomes through transport carriers or via early endosome remodelling to late endosomes (34). Rab5 is a marker of early endosomes and is known to be important in promoting endosome fusion in endocytosis, mediated by its guanine nucleotide exchange factor (GEF) that acts a molecular switch converting Rab5-GDP to Rab5-GTP (35). Late endosomes are formed by conversion of mature early endosomes, marked by the loss of Rab5 (early endosome) and acquisition of Rab7 (late endosome) (34). Data from our 210-gene siRNA screening work identified Rab5C as important in ASO productive uptake. Rab5C has also recently been reported to be important in the productive trafficking pathway of 2′MOE ASOs in engineered Stabilin-expressing cell lines, also confirming the findings from our siRNA screen (36). Our study suggests this role is also conserved in cancer cell lines and with cEt-ASOs. We demonstrate that loss of *RAB5C* mRNA interferes with the productive trafficking pathway of ASO, as we see a reduction in ASO-mediated *KRAS* down-regulation. This indicates that Rab5C may play an important role in mediating mechanisms of ASO escape from the early endosome, or ASO escape during early endosome maturation. Whilst we did not see any correlation between *RAB5C* expression and degree of ASO uptake (Figure 10C), differences in uptake may not be driven by overall Rab5C expression, but rather by functional recruitment that can be regulated by other factors. Rab5 has three distinct isoforms that share roughly 90% sequence homology (37,38). Preliminary screening in NCI-H2030 cells suggested a role for all three isoforms in the productive uptake of ASOs; however, *RAB5C* knockdown had the most robust impact on ASO productive uptake across moderate uptake cells H2030 and A549, likely due to its higher relative expression. Furthermore, analysis across the NSCLC cell panel and TCGA patient data also highlighted differential expression of *RAB5* isoforms with *RAB5C* being the most predominantly expressed. The Rab5C isoforms have also been shown to have distinct functions in EGFR degradation and trafficking (39,40) and Rab5C has also been reported to be associated with enhanced cellular motility and growth-factor activation of RAC1 (37). Therefore, it is also possible that there is an independent role for Rab5C in ASO trafficking and delivery distinct from Rab5A and Rab5B. Expanding the study across more cell lines would be interesting in determining the role of Rab5C and other isoforms in ASO productive trafficking outside of NSCLC.

### ASO traffics rapidly to the late endosome and lysosome in poor productive uptake cell lines and to the nucleus in cell with good productive uptake

**Figure 11.**
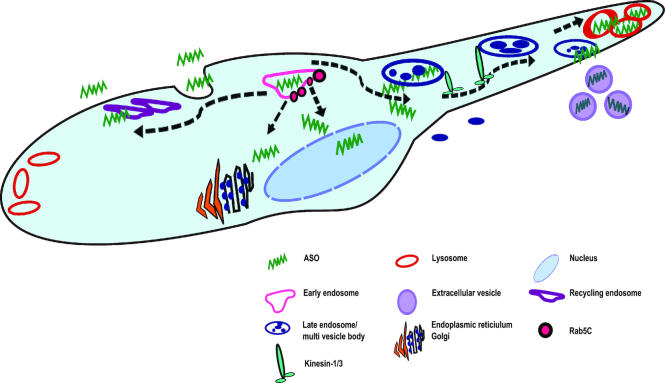
Schematic to show the proposed mechanism of ASO productive and non-productive trafficking in the cell. ASO is trafficked to the early endosome where it may be recycled back to the membrane or undergo endosomal escape mediated by Rab5C. The early endosome can fuse with the late endosome or multivesicular body and transport cargo, likely through motor proteins kinesin-1 and-3, either to the lysosome for degradation or to the cell periphery where it fuses with the plasma membrane and is secreted out of the cell via extracellular vesicles.

It is known that once cargo is contained within the late endosome a number of factors can affect its fate within the cell, such as acidification of the vesicle compartments required for degradation in the lysosome. Besides delivering cargo to the lysosome, late endosomes and lysosomes can also fuse with the plasma membrane delivering material out of the cell, known to be regulated by Rab27A (41–43). Across our cell line panel, we observed that trafficking of ASO to the late endosome and lysosome was more rapid in cells with poor productive uptake, suggesting that ASO may be rapidly degraded in these cell lines or bypass routes leading to productive uptake. We also observed a greater frequency of larger vesicles clustering in the poor productive uptake cell lines compared to the good uptake cell lines and these larger clusters of AZD4785 appeared to be MVBs (data not shown). Interestingly, in this study, cell lines that had poor ASO-mediated *KRAS* mRNA knockdown also had varied distribution of internalized ASO, localizing to the cell periphery. Our analysis identified differential expression of *KIF1A* and *KIF5C* encoding components of the kinesin motor proteins: kinesin-1 and kinesin-3. These motor proteins have previously been reported to participate in peripheral lysosomal trafficking along the microtubules (44). This suggests a mechanism by which ASOs are rapidly transported to the cell periphery by kinesin-1 and -3 in the cells with poor ASO uptake, leading to either degradation or exocytosis (Figure 11). It is well established that MVBs can fuse with the plasma membrane, resulting in the release of extracellular vesicles (45,46). Therefore, it is possible that the peripheral distribution of ASO is indicative of a functional pathway of exocytosis, whereby the MVBs are secreting intraluminal vesicles containing ASO out of the cell as exosomes. Further work investigating pathways of exocytosis and exosome secretion would be a key to understanding the non-productive trafficking pathways of ASOs within the cell.

In this work, we demonstrate a novel method for detecting intact ASO within the cell, culture media and cellular fractions. Our findings suggest that in cells with good ASO uptake we see an increase in ASO accumulation in the nucleus that is significantly higher compared to poor uptake cells. Comparison of total cell lysates showed comparable quantities of ASO between different cell lines, again confirming the differences in trafficking pathways between the good and poor productive uptake models.

## CONCLUSION

This study focused on cEt-modified ASOs, and is the first to investigate ASO uptake across a panel of clinically relevant cell lines, to characterize differences in productive uptake mechanisms. We have shown a comprehensive analysis of ASO driven target knockdown, kinetics and trafficking. In addition we have identified a possible mechanism through kinesin motor proteins that may be crucial for non-productive ASO trafficking to late endosome and lysosomes. Furthermore, we have presented novel insights into Rab5C-dependent trafficking of ASOs, which may determine the fate of the antisense oligonucleotide within the cell (Figure 11).

A number of recent studies have demonstrated mechanisms of 2′MOE ASO trafficking within the cell, concentrating on escape/release into the cellular cytoplasm (18,19). However, to date there have been no studies characterizing trafficking of therapeutic ASOs containing 2′-4′ constrained ethyl advanced chemistry modifications. We broadened our study beyond AZD4785 and characterized ASO uptake and intracellular distribution using two other ASO molecules including an ASO that contained both cEt and 2′MOE modifications. While our data suggest that mechanisms of ASO uptake and trafficking may be conserved across ASOs with different sequences and chemistry, further investigation across a broad range of ASOs including those with different mechanism of action would be interesting to determine if uptake mechanisms are conserved.

Whilst this study explores productive uptake of ASO across several clinically relevant cancer models, further work across other tumours types, *in vivo* models (e.g. in cell-derived and patient-derived xenograft (PDX) models), as well as normal cells and tissues, would help understand if Rab5C function in productive uptake is universally conserved. Finally, future work should explore exact molecular mechanisms engaged by kinesin proteins in non-productive uptake and Rab5C in facilitating productive uptake of ASOs. Continued work to establish mechanisms that may mediate ASO escape from the endosome into the cytoplasm or factors that may impair this will help identify patient selection strategies or strategies to enhance the pharmacological effects of antisense drugs.

## Supplementary Material

Supplementary Data
