# Supplementary material for: Differential uptake, kinetics and mechanisms of intracellular trafficking of next-generation antisense oligonucleotides across human cancer cell lines
Source: Nucleic Acids Res. 2019 Mar 30;47(9):4375–92. doi: 10.1093/nar/gkz214 (PMC6511877; doi:10.1093/nar/gkz214)
Supplement: Supplementary Data [file gkz214_supplemental_file.pdf]

Supplementary Figures

A

Compound	Mode	Parent ion	Daughter ion	Dwell	Cone Voltage	Collision Energy
**AZD4785**	ESI -ve	773	360.5	0.329	20	30

B

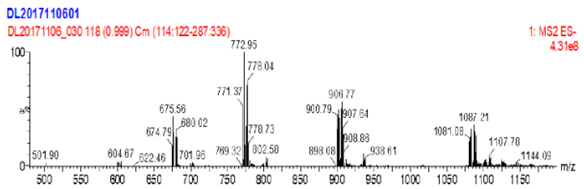

C

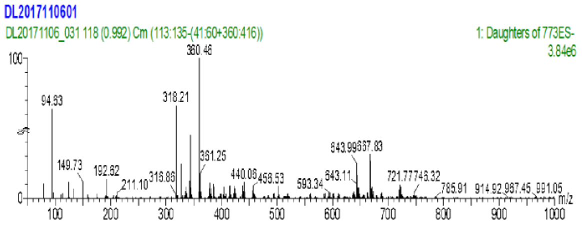

D

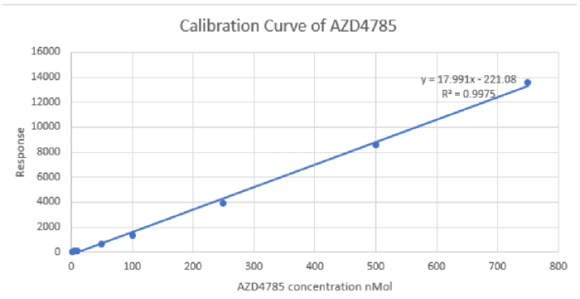

E

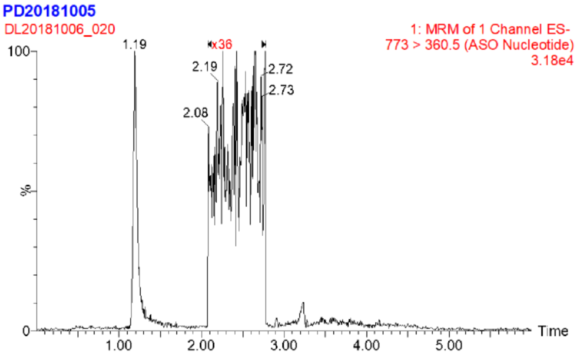

F

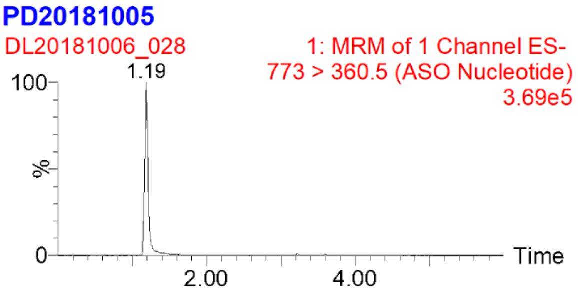

Supplementary Figure 1. Details of Mass spectrometry analysis of AZD4785 levels

Samples were analysed by UPLC-MS utilising a Waters Xevo TQ-XS (WBA0259) and an Acquity UPLC system from Waters consisting of Sample Manager (M16UFL953M), Acquity PDA (F17UPD457A), Column Oven (E17CMP703G) and Binary Solvent Manager (E17BUR621G). (A) The Waters TQ-XS was operated in -ve ion Electrospray (ESI) mode with the optimised transitions for AZD4785 (utilising the M-7H ion from the charge envelope). The chromatograms at each transition were extracted, smoothed and integrated to give the standard curve chromatograms of the samples were treated similarly and by linear regression (1/x) an in-cell concentration was established in the re-suspended cell lysate using the Waters MassLynx TargetLynx™ product. The mean r^2 was observed to be >0.991 and the mean error of the QC samples of 11.5%. (B) Parent and (C) daughter ion spectra from infusion of 10 µM stock solution in DMSO. (D) Representative standard curve of AZD4785 (as generated using TargetLynx™). (E) Representative chromatograms Showing S/N ratio of AZD4785 at 50nM. (F) Representative chromatography of AZD4785.

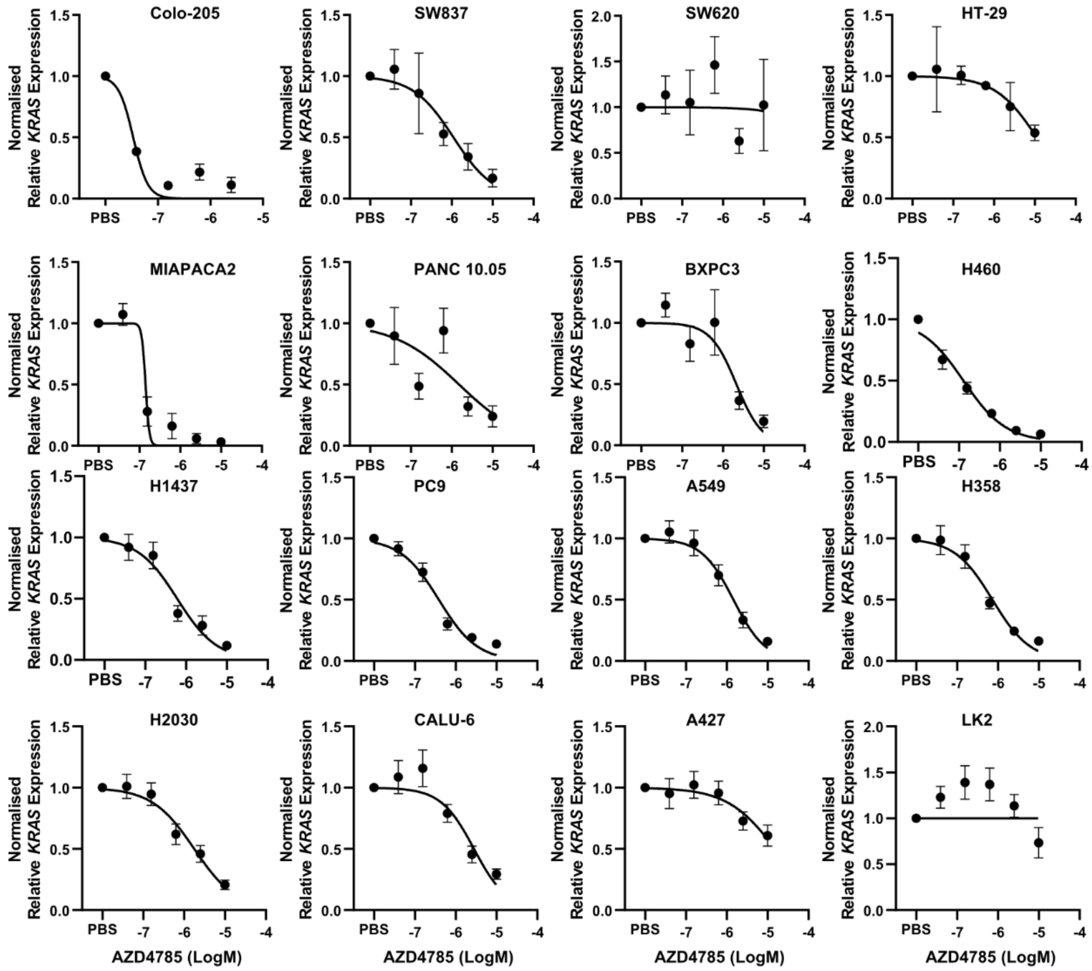

Supplementary Figure 2. Knockdown of *KRAS* mRNA by AZD4785 across a panel of cancer cells
Cells were treated with a dose range of AZD4785 for 72 hours before RT-qPCR was carried out to assess *KRAS* mRNA levels. Data was normalised to the housekeeping gene *18S rRNA* and PBS control. Full IC_{50} dose-response curves are shown as a mean from at least two experiments; error bars represent standard error of the mean (SEM).

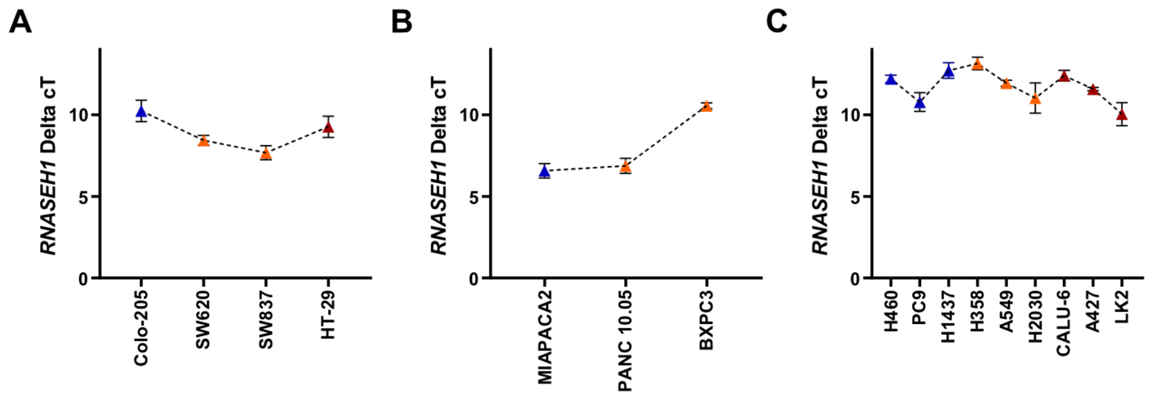

Supplementary Figure 3. Basal *RNASEH1* mRNA levels across a panel of cancer cells
RT-qPCR was carried out to assess basal *RNASEH1* mRNA levels across (A) colon, (B) pancreatic and (C) non-small cell lung cancer cell lines. Data was normalised to the housekeeping gene *18S rRNA*. Data shown represents the mean, error bars represent standard error of the mean (SEM).

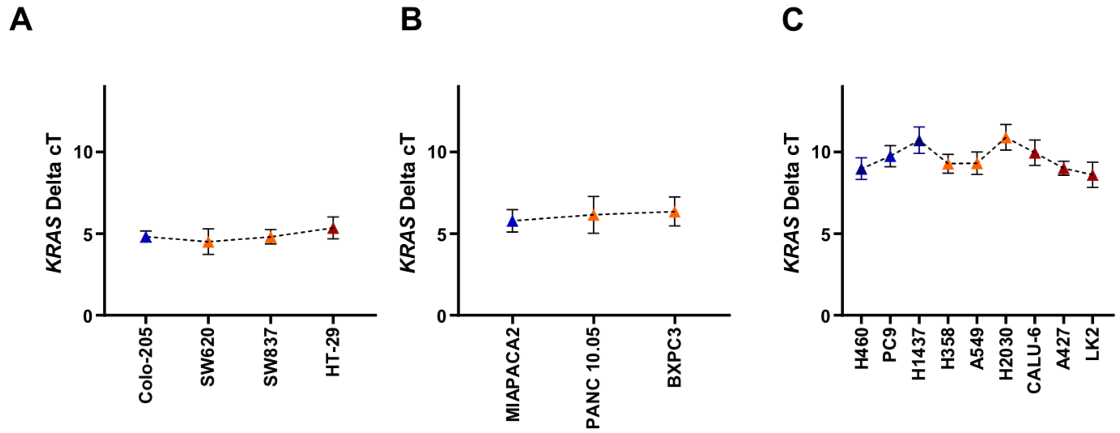

Supplementary Figure 4. Basal *KRAS* mRNA levels across a panel of cancer cells
RT-qPCR was carried out to assess basal *KRAS* mRNA levels across (A) colon, (B) pancreatic and (C) non-small cell lung cancer cell lines. Data was normalised to the housekeeping gene *18S rRNA*. Data shown represents the mean of minimum three experiments, error bars represent standard error of the mean (SEM).

Tukey's multiple comparisons test	Significance	Adjusted P Value
Colo-205 vs. SW620	***	0.0005
Colo-205 vs. LK2	*	0.0111
SW837 vs. SW620	**	0.0015
SW837 vs. LK2	*	0.0388
SW620 vs. MIAPACA2	****	<0.0001
SW620 vs. PANC 10.05	**	0.0058
SW620 vs. BXPC3	**	0.0026
SW620 vs. H460	****	<0.0001
SW620 vs. PC9	****	<0.0001
SW620 vs. H1437	****	<0.0001
SW620 vs. H358	****	<0.0001
SW620 vs. A549	****	<0.0001
SW620 vs. H2030	***	0.0003
SW620 vs. CALU-6	**	0.0019
MIAPACA2 vs. A427	*	0.0212
MIAPACA2 vs. LK2	**	0.002
H460 vs. A427	***	0.0008
H460 vs. LK2	****	<0.0001
PC9 vs. A427	**	0.0078
PC9 vs. LK2	***	0.0003
H1437 vs. A427	*	0.0119
H1437 vs. LK2	***	0.0006
H358 vs. A427	*	0.0149
H358 vs. LK2	***	0.0006
A549 vs. A427	*	0.0211
A549 vs. LK2	**	0.001
H2030 vs. LK2	**	0.007

Supplementary Table 1. Statistically signficant differences in maximum inhibition of *KRAS* mRNA knockdown by AZD4785 at between cell lines

Maximum inhibition of *KRAS* mRNA knockdown with 10 µM of AZD4785 at 72 hours was determined across cell lines (Figure 1B). ANOVA and Tukey's post-hoc test was carried out for multiple comparisons between cell lines and all cell lines with significance (adjusted P-value) are shown in the table.

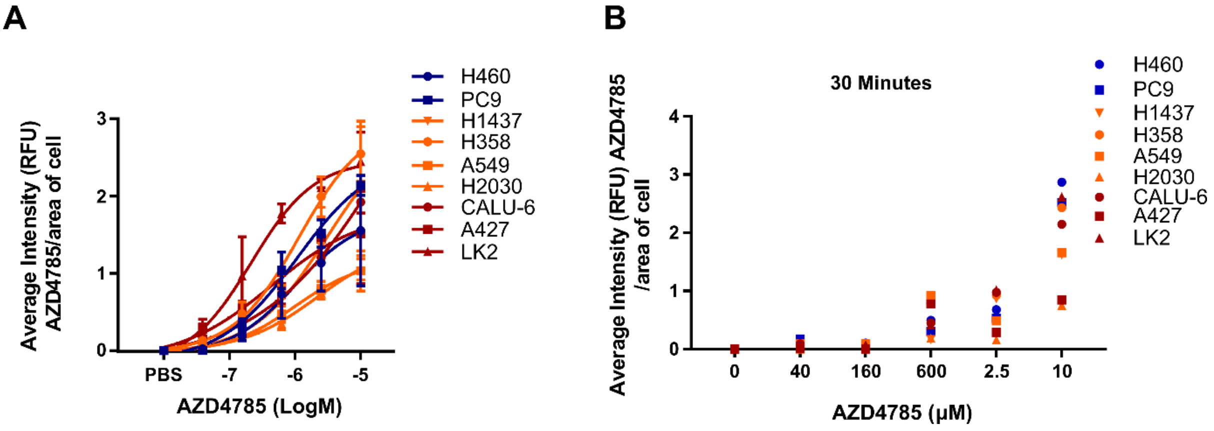

Supplementary Figure 5. **Internalisation of AZD4785 across NSCLC cell lines**

Cells were seeded onto glass-bottomed plates and treated with a dose range of AZD4785 or PBS. Cells were fixed at (A) 72 hours and (B) 30 minutes and nuclei were stained with Hoechst, cytoskeleton with alpha tubulin and AZD4785 with Rb anti-ASO antibody. Images were acquired and analysed using Columbus software and relative AZD4785 fluorescence (RFU) was quantified over 16 fields of view per well and normalised to the average cell area. Data were analysed from a minimum of three experiments, error bars represent SEM.

Cell Line	Dose AZD4785	Time Frame	Adjusted P Value
H460 vs LK2	10 µM	24 hours	0.0089
H460 vs A427	10 µM	24 hours	0.0221
PC9 vs LK2	10 µM	48 hours	0.0051
PC9 vs A427	10 µM	48 hours	0.0307
H358 vs LK2	10 µM	48 hours	0.0220
H460 vs LK2	10 µM	48 hours	0.0028
H460 vs A427	10 µM	48 hours	0.0160
H1437 vs LK2	10 µM	48 hours	0.0092
H1437 vs A427	10 µM	48 hours	0.0482
A549 vs LK2	10 µM	48 hours	0.0154
Calu-6 vs LK2	10 µM	48 hours	0.0478
PC9 vs A427	2.5 µM	24 hours	0.0441
H460 vs A549	2.5 µM	24 hours	0.0107
H460 vs LK2	2.5 µM	24 hours	0.0065
H460 vs A427	2.5 µM	24 hours	0.0023
PC9 vs LK2	2.5 µM	48 hours	0.0005
PC9 vs A427	2.5 µM	48 hours	0.0373
H2030 vs LK2	2.5 µM	48 hours	0.0492
H358 vs LK2	2.5 µM	48 hours	0.0030
H460 vs LK2	2.5 µM	48 hours	0.0002
H460 vs A427	2.5 µM	48 hours	0.0175
H1437 vs LK2	2.5 µM	48 hours	0.0012
A549 vs LK2	2.5 µM	48 hours	0.0094
Calu-6 vs LK2	2.5 µM	48 hours	0.0315
H460 vs A549	0.6 µM	24 hours	0.0146
H460 vs LK2	0.6 µM	24 hours	0.0031
H1437 vs LK2	0.6 µM	24 hours	0.0251
PC9 vs LK2	0.6 µM	48 hours	0.0095
H460 vs LK2	0.6 µM	48 hours	0.0105
PC9 vs LK2	0.16 µM	48 hours	0.0416
H2030 vs H460	0.16 µM	48 hours	0.0358
H358 vs LK2	0.16 µM	48 hours	0.0484
H460 vs LK2	0.16 µM	48 hours	0.0181

Supplementary Table 2. Statistically signficant differences in *KRAS* mRNA knockdown by AZD4785 between cell lines across dose and time

NSCLC cell lines were treated with a dose range of AZD4785 and washed after various time points before fixing and processing at 72 hours (Figure 4A). A one-way analysis of variance (ANOVA) was performed to assess significance. Tukey's post-hoc test was carried out for multiple comparisons between cell lines and all cell lines with significance (adjusted P-value) are shown in the table.

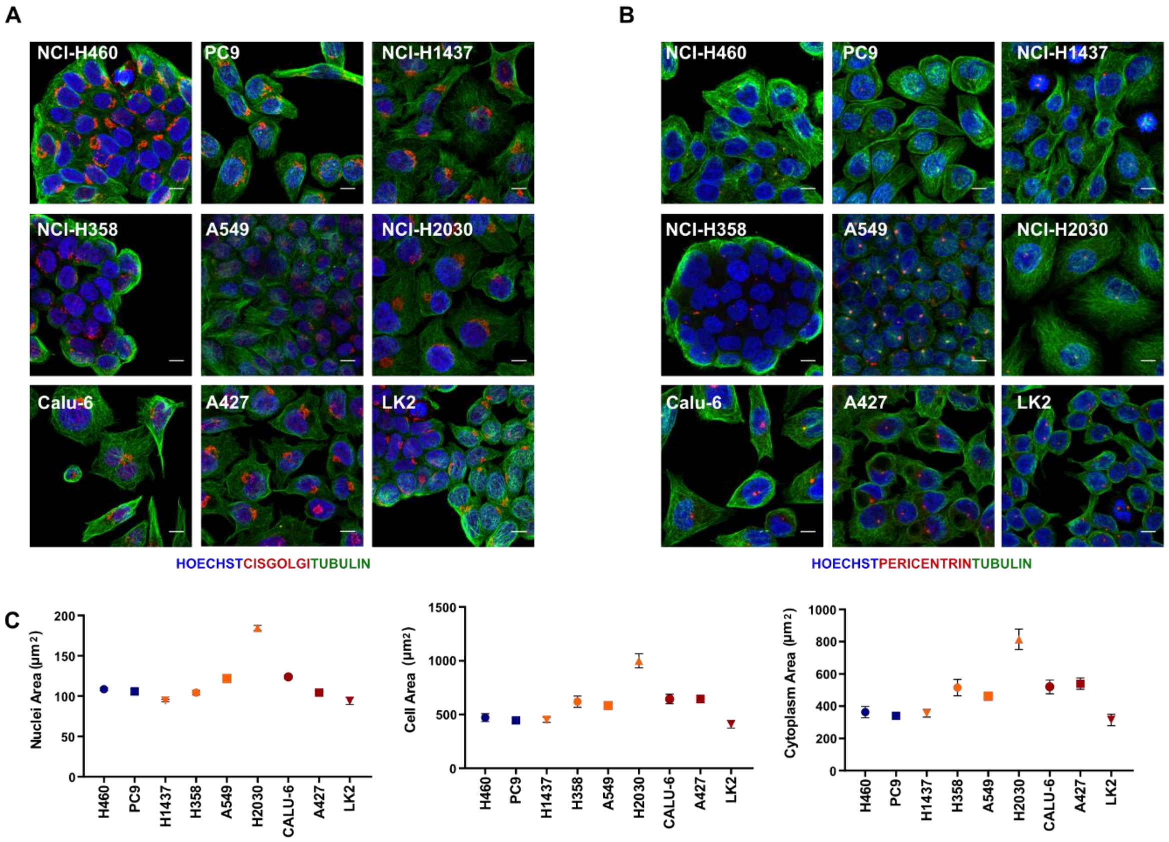

Supplementary Figure 6. **Organisation of cellular organelles is varied between good, moderate and poor AZD4785 uptake cell lines**

Cell lines were seeded onto glass-bottomed plates and fixed after 24 hours. Nuclei were stained with Hoechst, cytoskeleton with alpha tubulin antibody and (A) Golgi with cis-Golgi antibody (B) centrosomes with pericentrin antibody. Z- stack images were acquired and images shown as maximum projection, scale bar 10 µm. (C) Cell properties were analysed from cells fixed at various time points, images were taken across 16 fields of view, six wells per cell line, and data analysed using Columbus Software (Perkin Elmer). Mean area of nuclei, cell and cytoplasm are shown, error bars represent SEM.

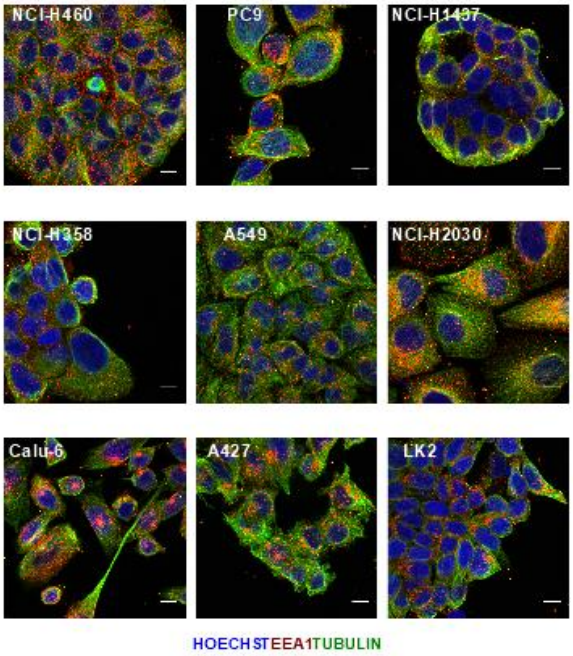

Supplementary Figure 7. **Distribution of Early Endosome across NSCLC cells**
Cell lines were seeded onto glass-bottomed plates and fixed after 24 hours. Nuclei were stained with Hoechst, cytoskeleton with alpha tubulin antibody and early endosome with EEA1 antibody. Z- stack images were acquired and images shown as maximum projection, scale bar 10 µm.

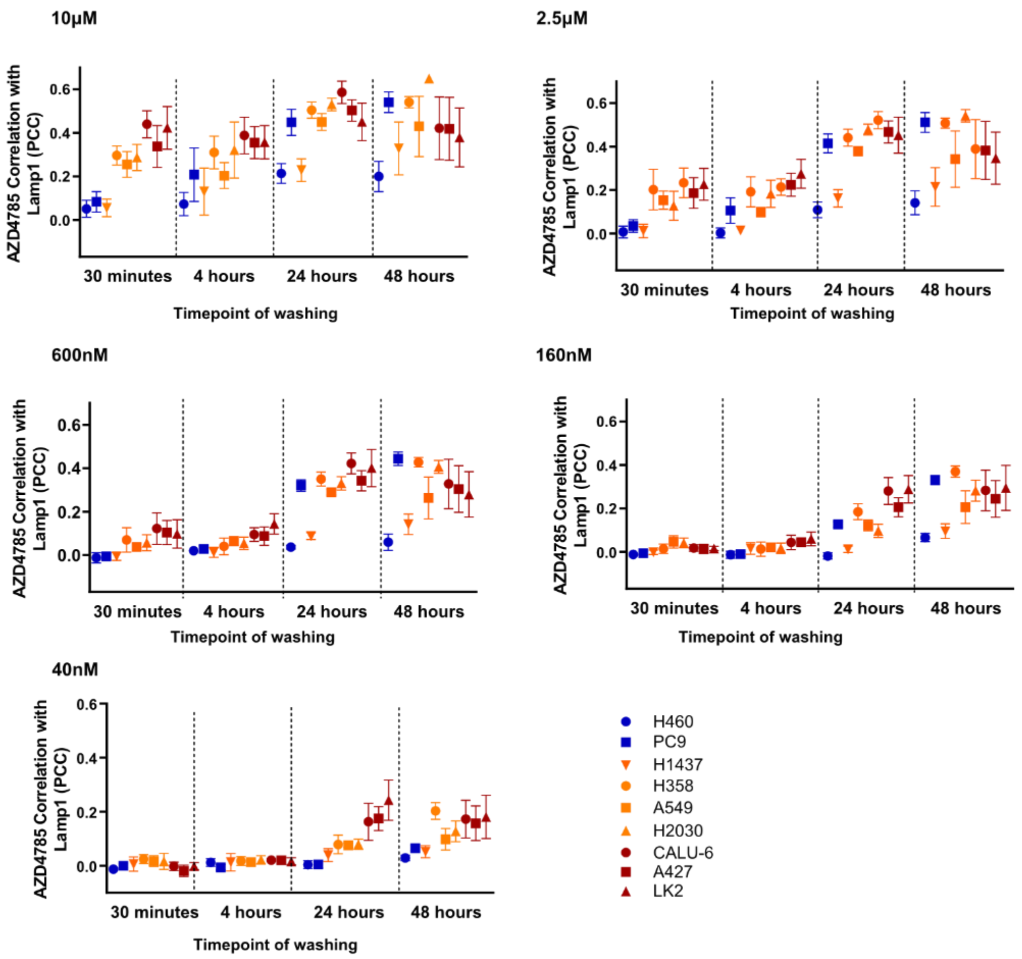

Supplementary Figure 8. **Co-localisation of AZD4785 and lysosome marker LAMP-1 across NSCLC cell lines**

Cell lines were seeded onto glass-bottomed plates and treated with a dose range of AZD4785 or PBS. Cells were washed at various time points and fixed at 72 hours (Figure 4A), then nuclei were stained with Hoechst, cytoskeleton with alpha tubulin antibody and AZD4785 with the anti-ASO antibody. Images were acquired and analysed using Columbus Software (Perkin Elmer). Pearson's correlation coefficient (PCC) was quantified per cell over 16 fields of view per well. Data was analysed from a minimum of three experiments. Error bars represent SEM.

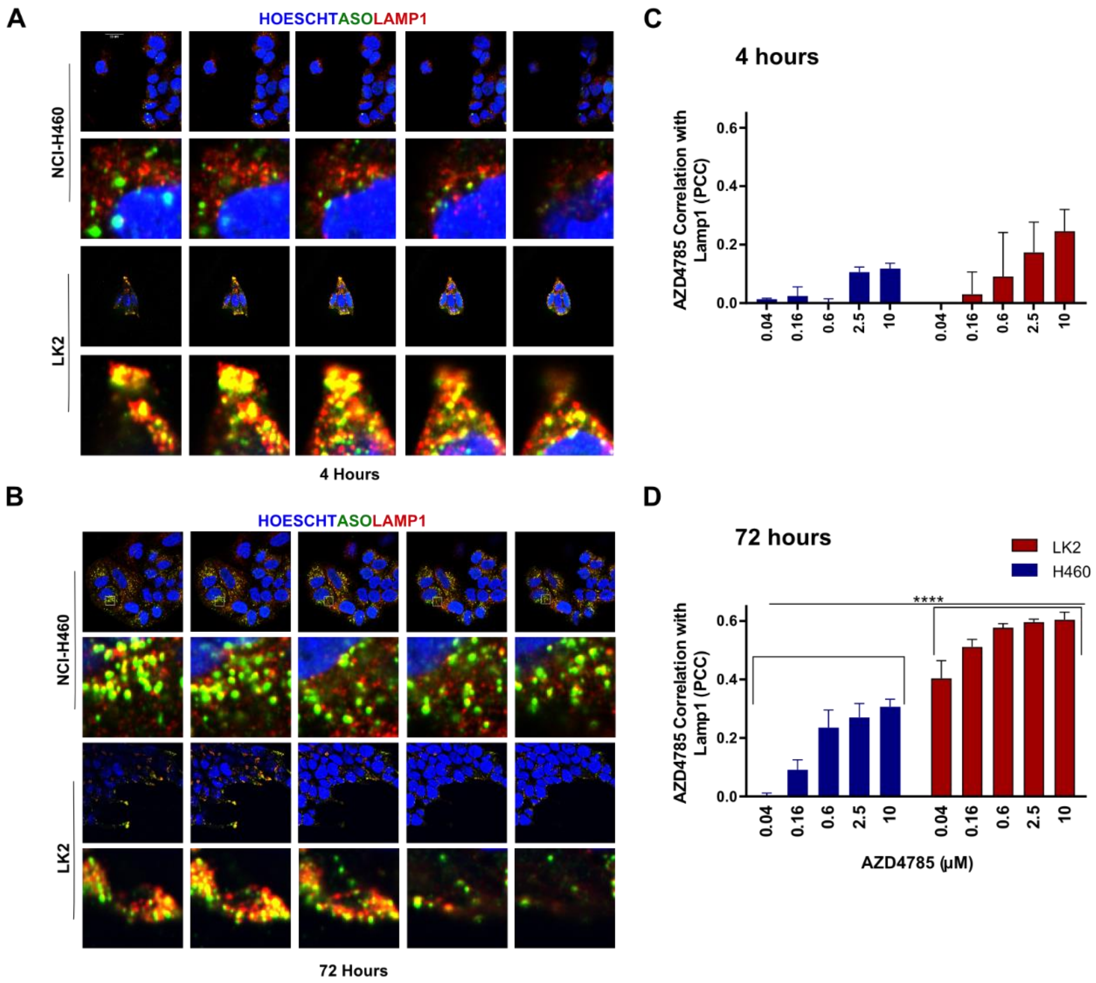

Supplementary Figure 9. AZD4785 shows strong and rapid co-localisation with the lysosome in cells which have poor *KRAS* knockdown

(A and B) Individual confocal z-stack images of a representative field of NCI-H460 and LK2 cells to visualise AZD4785 (green) and LAMP-1 (red) co-localisation. Images taken at (A) four hours and (B) 72 hours following treatment with 10µM of AZD4785. The enlarged image indicates 10x zoom, scale bar 20µm. (C and D) Quantification of co-localisation between AZD4785 and LAMP-1 in NCI-H460 cells and LK2 cells calculated by Pearson's correlation coefficient at (C) 4-hour and (D) 72-hour time points on images taken across one plane, from 16 fields of view per well. Data shown is mean from a minimum of three experiments. Error bars represent SEM and multiple t-tests were carried out ($p<0.001$).

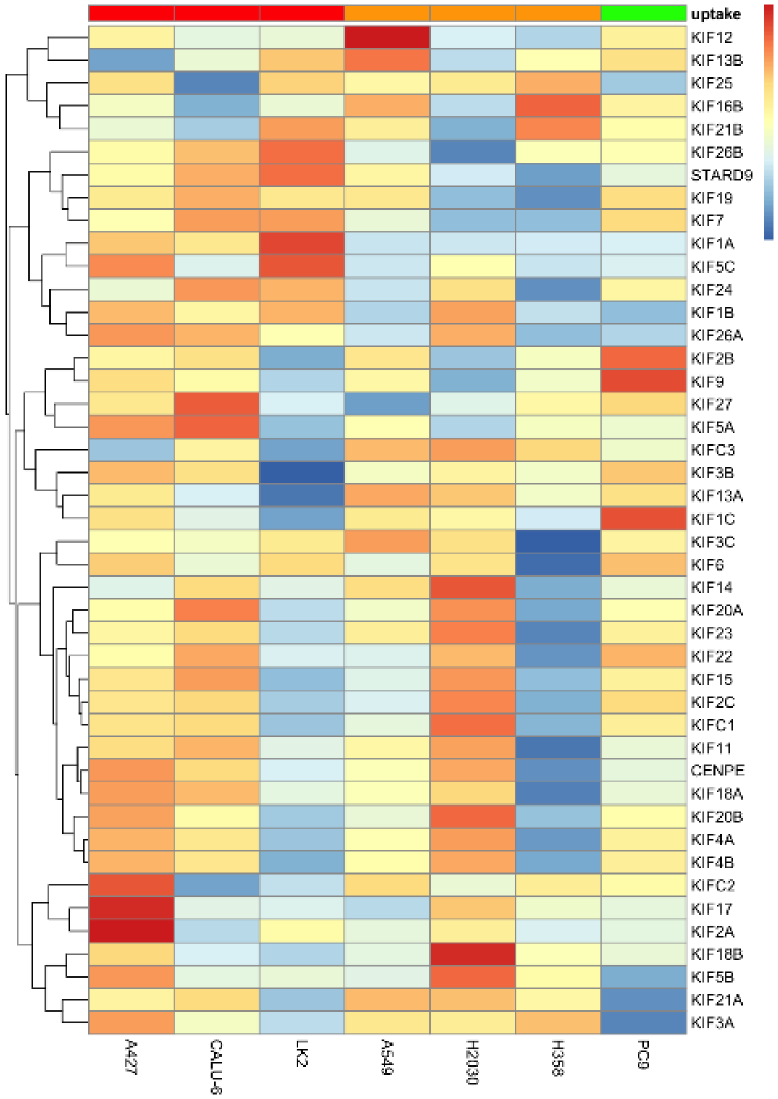

Supplementary Figure 10. Expression of kinesin motor proteins across the panel of cell lines

Gene expression signatures from NSCLC cells were analysed using OmicSoft ArraySuite, Oncoland Software, with RNAseq expression data from Sanger_38 database. Log_2 (FPKM+0.1) values were plotted for the kinesin gene family as a heatmap.

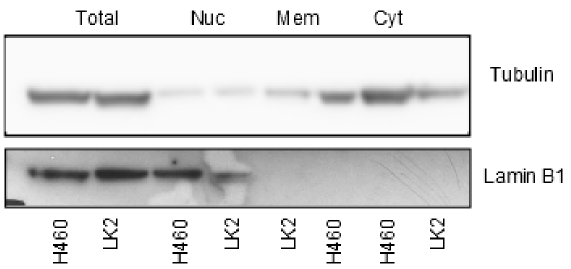

Supplementary Figure 11. Western blot analysis of lysates from cell fractionation
LK2 and NCI-H460 cells were fractionated and used for mass spectrometry experiments to detect full length intact ASO (Figure 8). Western blotting was carried out to confirm purity of nuclear fraction as lamin B1 is specific to the nucleus.

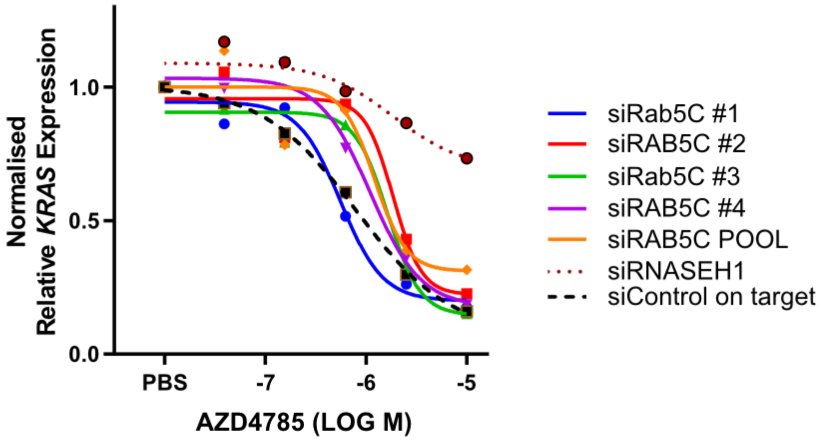

Supplementary Figure 12. Validation of *RAB5C* in the productive uptake of AZD4785 in NCI-H2030 cells
Cells were transfected with pools or individual siRNA targeting *RAB5C*, *RNASHE1* or a non-targeting control for 72 hours before treating with a dose range of AZD4785. RT-qPCR was carried out to determine *KRAS* knockdown and IC_{50} dose-response curves were plotted. Data were normalised to *18S rRNA* and shown relative to PBS control.

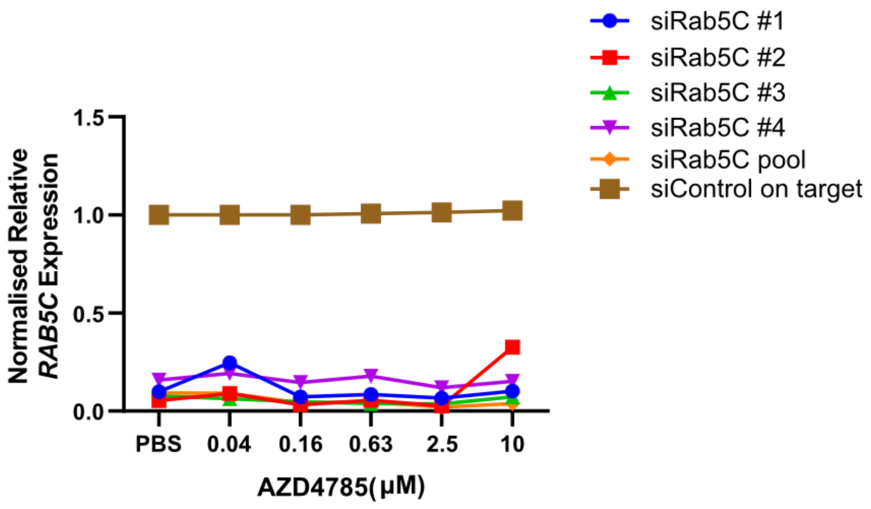

Supplementary Figure 13. Validation of *RAB5C* knockdown AZD4785 in NCI-H2030 cells
Cells were transfected with pools or individual siRNA targeting *RAB5C* or a non-targeting control for 72 hours before treating with a dose range of AZD4785. Knockdown of *RAB5C* was confirmed by RT-qPCR. Data were normalised to *18S rRNA* and shown relative to the non targeting control.

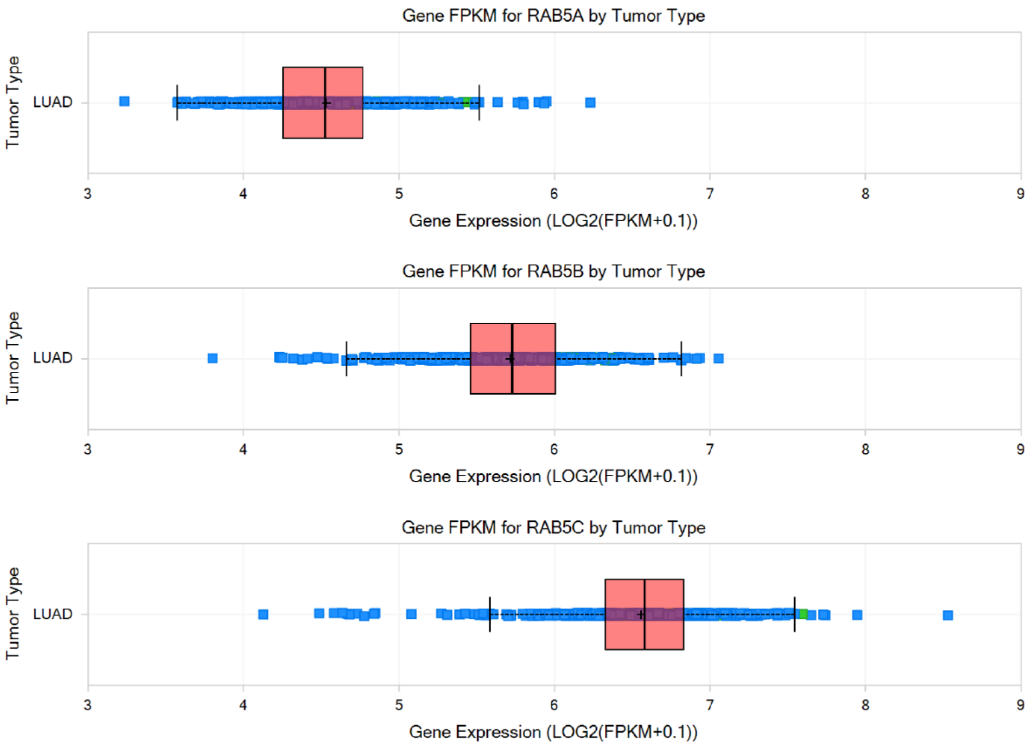

Supplementary Figure 14. *RAB5* isoform expression across lung tumours
Patient lung adenocarcinoma data for the expression of *RAB5* isoforms from the TCGA_37 database were analysed using OmicSoft ArraySuite, OncoLand software. Log_2 (FPKM+0.1) values were plotted for *RAB5A, RAB5B* and *RAB5C*.
